# Detection of Pleiotropy through a Phenome-Wide Association Study (PheWAS) of Epidemiologic Data as Part of the Environmental Architecture for Genes Linked to Environment (EAGLE) Study

**DOI:** 10.1371/journal.pgen.1004678

**Published:** 2014-12-04

**Authors:** Molly A. Hall, Anurag Verma, Kristin D. Brown-Gentry, Robert Goodloe, Jonathan Boston, Sarah Wilson, Bob McClellan, Cara Sutcliffe, Holly H. Dilks, Nila B. Gillani, Hailing Jin, Ping Mayo, Melissa Allen, Nathalie Schnetz-Boutaud, Dana C. Crawford, Marylyn D. Ritchie, Sarah A. Pendergrass

**Affiliations:** 1 Center for Systems Genomics, Department of Biochemistry and Molecular Biology, The Huck Institutes of the Life Sciences, The Pennsylvania State University, University Park, Pennsylvania, United States of America; 2 Center for Human Genetics Research, Vanderbilt University, Nashville, Tennessee, United States of America; 3 Department of Molecular Physiology and Biophysics, Vanderbilt University, Nashville, Tennessee, United States of America; Georgia Institute of Technology, United States of America

## Abstract

We performed a Phenome-wide association study (PheWAS) utilizing diverse genotypic and phenotypic data existing across multiple populations in the National Health and Nutrition Examination Surveys (NHANES), conducted by the Centers for Disease Control and Prevention (CDC), and accessed by the Epidemiological Architecture for Genes Linked to Environment (EAGLE) study. We calculated comprehensive tests of association in Genetic NHANES using 80 SNPs and 1,008 phenotypes (grouped into 184 phenotype classes), stratified by race-ethnicity. Genetic NHANES includes three surveys (NHANES III, 1999–2000, and 2001–2002) and three race-ethnicities: non-Hispanic whites (n = 6,634), non-Hispanic blacks (n = 3,458), and Mexican Americans (n = 3,950). We identified 69 PheWAS associations replicating across surveys for the same SNP, phenotype-class, direction of effect, and race-ethnicity at p<0.01, allele frequency >0.01, and sample size >200. Of these 69 PheWAS associations, 39 replicated previously reported SNP-phenotype associations, 9 were related to previously reported associations, and 21 were novel associations. Fourteen results had the same direction of effect across more than one race-ethnicity: one result was novel, 11 replicated previously reported associations, and two were related to previously reported results. Thirteen SNPs showed evidence of pleiotropy. We further explored results with gene-based biological networks, contrasting the direction of effect for pleiotropic associations across phenotypes. One PheWAS result was *ABCG2* missense SNP rs2231142, associated with uric acid levels in both non-Hispanic whites and Mexican Americans, protoporphyrin levels in non-Hispanic whites and Mexican Americans, and blood pressure levels in Mexican Americans. Another example was SNP rs1800588 near *LIPC*, significantly associated with the novel phenotypes of folate levels (Mexican Americans), vitamin E levels (non-Hispanic whites) and triglyceride levels (non-Hispanic whites), and replication for cholesterol levels. The results of this PheWAS show the utility of this approach for exposing more of the complex genetic architecture underlying multiple traits, through generating novel hypotheses for future research.

## Author Summary

The Epidemiological Architecture for Genes Linked to Environment (EAGLE) study performed a Phenome-Wide Association Study (PheWAS) to investigate comprehensive associations between a wide range of phenotypes and single-nucleotide polymorphisms using the diverse genotypic and phenotypic data that exists across multiple populations in the National Health and Nutrition Examination Surveys (NHANES), conducted by the Centers for Disease Control and Prevention (CDC). In this study, we replicated known genotype-phenotype associations, identified genotypes associated with phenotypes related to previously reported associations, and most importantly, identified a series of novel genotype-phenotype associations. We also identified potential pleiotropy; that is, SNPs associated with more than one phenotype. We explored the features of these PheWAS results, characterizing any potential functionality of the SNPs of this study, determining association results that were found in more than one racial/ethnic group for the same SNP and phenotype, identifying novel direction of effect relationships for SNPs demonstrating potential pleiotropy, and investigating the association results in the context of gene-based biological networks. Through considering the SNP associations on multiple phenotypic outcomes, as well as through exploring pleiotropy, we may be able to leverage the results of PheWAS to uncover more of the complex underlying genomic architecture of complex traits.

## Introduction

Genome-wide association studies (GWAS) have led to the discovery of thousands of variants associated with disease and phenotypic outcomes [1]. GWAS focus on investigating the association between hundreds of thousands to over a million single nucleotide polymorphisms (SNPs) and a single, or small set, of phenotypes and/or disease outcomes. While a wealth of information about the relationship between SNPs and phenotypes has been revealed, an extensive picture of the complex genetic architecture underlying common disease has yet to be elucidated. In addition, the relationship between SNPs and multiple phenotypes (pleiotropy) is only beginning to be explored.

A complementary approach to GWAS are phenome-wide association studies (PheWAS), an approach for investigating the complex networks that exist between human phenotypes and genetic variation, through testing a series of SNPs for association with a large and diverse set of phenotypes [2]–[5]. These analyses can be used to investigate the relationship between genetic variants and presence/absence of disease and phenotypic outcomes as well as the association between genetic variation and intermediate clinically measured variables such as cholesterol levels, blood pressure measurements, and total iron binding capacity. PheWAS can be used to replicate relationships found in GWAS as well as to discover novel associations and generate hypotheses for further research. This approach also allows for the detection of SNPs with pleiotropic effects, where one genetic variant is associated with multiple phenotypes [6], [7]. Investigating the interrelationships that exist between phenotypes as well as between genetic variation and phenotypic variation has the potential for uncovering the complex mechanisms underlying common human phenotypes.

Here we describe a PheWAS using epidemiologic data from the National Health and Nutrition Examination Surveys (NHANES) collected by the Centers for Disease Control and Prevention and accessed by the Epidemiological Architecture for Genes Linked to Environment (EAGLE) study as part of the Population Architecture using Genomics and Epidemiology (PAGE) network [8]. A major focus of the PAGE network is the replication and generalization of GWAS-identified variants in diverse populations, as the majority of published GWAS have been performed in populations of European-descent with little generalization across other racial/ethnic groups. Thus, the PAGE network has pursued investigating associations for genetic variants that have been well replicated in previous research across ancestry groups beyond European-descent.

As a part of PAGE, EAGLE genotyped 80 GWAS-identified variants in two NHANES datasets representing three surveys: *NHANES III*, collected between 1991 and 1994, and *Continuous NHANES* which was collected between 1999–2000 and 2001–2002 across three race-ethnicities. The majority of the SNPs within our study were chosen for genotyping based on published lipid trait genetic association studies (51 SNPs), but our study also included SNPs previously associated with phenotypes such as C-reactive protein levels, coronary heart disease, and age-related macular degeneration, with detailed information about these SNPs in S1 Table. Genotyping was performed in a total of 14,998 NHANES participants with DNA samples including 6,634 self-reported non-Hispanic whites, 3,458 self-reported non-Hispanic blacks, and 3,950 self-reported Mexican Americans. Similar to the PheWAS framework outlined by the PAGE study [3], we performed comprehensive unadjusted tests of association for 80 SNPs with 1,008 phenotypes, using linear or logistic regression, depending on the phenotype, stratified by race-ethnicity.

With this approach we replicated many previously reported associations and identified novel genotype-phenotype relationships. We have performed our analyses across multiple genetic ancestries. Most importantly, we have also found indications of pleiotropy for a number of the SNPs included in our investigation. Contrasting the association results for SNPs with multiple phenotypes, interesting direction of effect differences were identified. We further explored the relationship between SNPs, genes, and known biological relationships between the genes, identifying network relationships within these results. The findings in this paper demonstrate that PheWAS is a useful method for both validating findings from GWAS and discovering previously unknown genotype-phenotype relationships in diverse populations, enriching our understanding of the complex underpinnings of human phenotypes.

## Results

The study population characteristics for the epidemiologic surveys accessed by EAGLE for this PheWAS are given in Table 1. Across the data collected for NHANES, there were 14,998 participants with DNA samples. More than half of the participants were female (54.12%), and the median age was 43. While ∼44% of the samples were from participants self-described as non-Hispanic white (n = 6,634), more than half of the samples were from participants self-described as either non-Hispanic black (n = 3,458) or Mexican American (n = 3,950). As expected, based on ascertainment and changes in consenting for genetic studies [9], NHANES III had more female and non-European participants with DNA samples compared with Continuous NHANES.

**Table 1:** Study population characteristics.

		NHANES III (n = 7,159)	Continuous NHANES (n = 7,839)	Combined NHANES (n = 14,998)
**% Female** *		56.67	51.79	54.12
**Median age**		38	47	43
	**NHW**	51	52	51
	**NHB**	33	45	38
	**MA**	33	43	38
**Race-ethnicity**				
	**% NHW**	36.74	51.07	44.23
	**% NHB**	29.45	17.22	23.06
	**% MA**	28.96	23.94	26.34
**Number of phenotypes**		750	258	489

Abbreviations: non-Hispanic white (NHW), non-Hispanic black (NHB), Mexican American (MA).

*χ^2^ = 35.95; p<0.0001.

**Figure 1:**
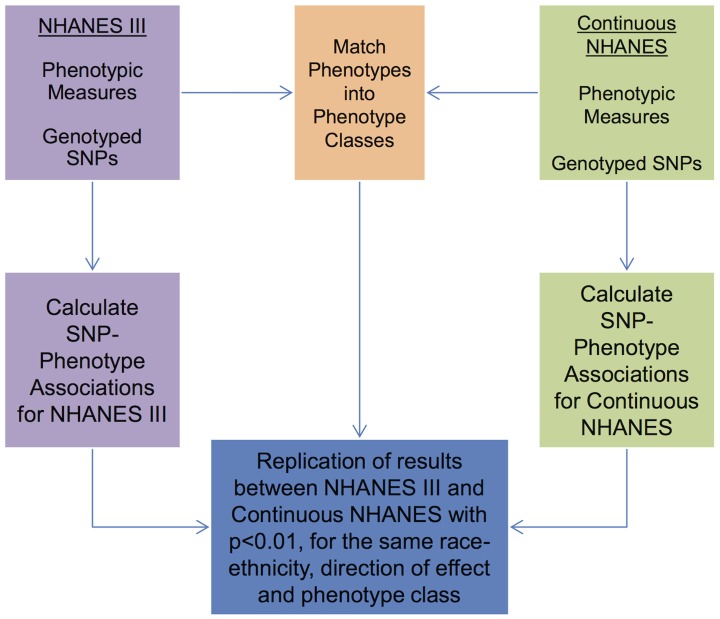
Overview of the approach for this study. Genotypic and phenotypic data were collected in NHANES III and Continuous NHANES. The phenotypes for the two studies were matched into phenotype classes. Comprehensive associations were calculated for the genotypes and phenotypes for each survey independently. The results that were found in both surveys, with p<0.01, for the same phenotype-class, and race-ethnicity, and same direction of effect, were maintained for further inspection in this study.

As detailed in the PheWAS workflow diagram shown in Fig. 1, we first identified 184 phenotype classes across NHANES from a total of 1,008 unique variables available for analysis in NHANES III and Continuous NHANES, respectively (Table 2). We then performed unadjusted single SNP tests of association assuming an additive genetic model for each SNP and phenotype (within each phenotype class) in NHANES III and Continuous NHANES. Our criteria for a significant PheWAS result was a SNP-phenotype association observed in both NHANES III and Continuous NHANES with p-value <0.01, for SNPs with an allele frequency >0.01, and a sample size >200, for the same race-ethnicity, phenotype-class, and direction of effect. We identified 69 PheWAS results meeting this significance threshold. Of these 69 PheWAS results, 39 replicated previously reported SNP-phenotype associations from the literature. Of the remaining results, 9 were related to previously reported associations in the literature, and 21 were novel SNP-phenotype associations. Moreover, 13 SNPs showed evidence of pleiotropy – where a particular SNP was associated with more than one phenotype. For the majority of results meeting our PheWAS criteria for replication, each SNP had multiple associations for each phenotype class; thus, in the text we report only the most statistically significant result. We detail all association results meeting our PheWAS criteria for replication in S2, S3, and S4 Tables and Table 3.

**Table 2:** Phenotype-classes.

**Anthropometry**	Body measurement (weight)	**Disease/Condition**	Allergy	**Exposures**	Alcohol	**Liver**	Liver
	Body measurements		Anxiety (symptoms)		Caffeine		Liver (albumin)
	Body measurements (arm)		Arthritis		Calcium intake		Liver (alkaline phosphatase)
	Body measurements (BMI)		Blindness		Caloric intake		Liver (bilirubin)
	Body measurements (head)		Broken/Fractured Bone		Carbohydrate intake		Liver (GGT)
	Body measurements (height)		Cataracts		Carotene intake	**Lung**	Asthma
	Body measurements (leg)		Cell/Tissue Damage		Cholesterol intake		Chest
	Body measurements (skinfold)		Cerebral Palsy		Copper intake		Chronic Bronchitis
	Body measurements (waist)		Chronic Limp		Fat intake		Lung
	Body measurements (weight)		Cold		Fiber intake		Lungs
**Blood Pressure**	Blood pressure		Depression (symptoms)		Folate intake	**Other**	Age
	Blood pressure (Ankle Brachial Pressure Index)		Diabetes		Folic acid intake		Alanine Aminotransferase Test
	Blood pressure (Brachial Systolic)		Diabetes (glycohemoglobin)		Iron intake		Aspartate Aminotransferase Test
	Blood pressure (Diastolic)		Emphysema		Magnesium intake		Balance Dental
	Blood pressure (Posterior tibial) systolip)		Epilepsy		Niacin intake		Dental Maximum Inflation Level
	Blood pressure (Systolic)		GoiterFracture		Nutrition		Urination
					Noise Exposure		Urine Test
	High blood pressure		Gout		Phosphorus intake	**Other Measurements**	Bleeding Disorder Test
	High blood pressure (diagnosis)		Hay fever		Potassium		Homocysteine
	Hypertension (doctor diagnosed) Recommendation)		Heart Attack		Protein intake		Hypoglycemia (C-peptide)
			Hay fever				
	Hypertension (medication)		Hepatitis Antibody		Riboflavin intake		Inflammation (C-reactive protein)
**Bone Density**	Bone density		Hip Dysplasia		Smoking		Osmolality
	Bone density (bone alkaline phosphatase)		Infection		Sodium intake		Protoporphyrin
	Bone density (calcium)		Injury		Thiamin intake		Total Iron Binding Content (TIBC)
	Bone density (N-telopeptides)		Leg Pain				Transferrin
**Circulating Cell Measurements**	Blood clotting		Lupus		Tobacco Measurement	**Reproductive**	Birth Control
	Erythrocyte protoporphyrin		Osteoporosis		Tocopherol intake		Breast Feeding
	Hematocrit		Pain		Vitamin		Fertility
	Hemoglobin		Paralysis		Vitamin A intake		Hysterectomy
	Monocyte		Rheumatic Fever		Vitamin B12 intake		Menstruation
	Monocytes		Rheumatoid Arthritis		Vitamin B6 intake		Oophorectomy
	Mononuclear number		Scoliosis		Vitamin C intake		Pregnancy
	Platelet		Stroke		vitamin D intake	**Thyroid**	Thyroid (T4)
	Red Blood Cell		Surgery		Vitamin E intake		Thyroid (TSH)
	White Blood Cell		Thyroid Disease		Vitamin K intake		
**Circulating Levels**	Bicarbonate		Tissue Damage		Water intake		
	Blood Test (Protein)		Ulcer/Sore		Zinc intake		
	Calcium			**Hearing**	Deafness		
	Carotene				Ear measurement		
	Chloride				Ear pressure		
	Ferritin				Hearing		
					Ear tube		
	Folate Levels			**Heart**	Heart		
	Glucose				Pulse		
	Insulin			**Kidney**	Kidney (Creatinine)		
	Iron Levels				Kidney (Globulin)		
	Phosphorus				Kidney (Urea nitrogen))		
	Potassium				Kidney (Uric acid)		
	Selenium			**Lipids**	Apolipoprotein		
	Sodium				Cholesterol		
	Total protein				Cholesterol (Doctor)		
	Vitamin A levels				HDL Cholesterol		
	Vitamin B12				High Cholesterol		
	Vitamin C levels				LDL Cholesterol		
	Vitamin D levels				Lipoprotein		
	Vitamin E levels				Triglycerides		
	Vitamin K levels						

The phenotype classes of this study, grouped here into broader categories.

**Table 3:** Novel results.

Closest Gene(s)	SNP	Chromosome	Position	Coded Allele	Previously Reported Phenotype (PAGE)	Previously Reported Phenotype (GWAS Catalog)	Pubmed ID	Phenotype Class	Race/Ethnicity	Study	Phenotype Description	P-value	Beta(SE)	Sample Size
BSND - PCSK9	rs11206510	1	55496039	T	LDL Cholesterol	Coronary heart disease	20864672	Globulin	MA	NHANES III	(ln +1)Serum globulin (g/dL)	0.0095	0.012(0.0046)	2023
						Myocardial infarction (early onset)	18193043				Serum globulin (g/dL)	0.0099	0.052 (0.020)	2023
						LDL cholesterol	19198609				(ln +1)Serum globulin: SI (g/L)	0.0097	0.015(0.0058)	2023
							21378990				Serum globulin: SI (g/L)	0.0099	0.523(0.20)	2023
							19060906				(ln +1)Serumglobulin(g/dL)	0.0095	0.012(0.0046)	2023
											Serumglobulin(g/dL)	0.0099	0.052(0.020)	2023
											(ln +1)Serumglobulin:SI(g/L)	0.0097	0.015(0.00582)	2023
											Serumglobulin:SI(g/L)	0.0099	0.52(0.20)	2023
										NHANES 1999–2002	(ln +1)Globulin (g/L)	0.0042	0.012(0.0062)	1871
											Globulin (g/L)	0.0062	0.56(0.20)	1871
											(ln +1)Globulin (g/dL)	0.0047	0.014(0.0049)	1871
											Globulin (g/dL)	0.0062	0.056(0.020)	1871
										NHANES Combined	(ln +1)Serum globulin (g/dL)	0.00092	0.011(0.0034)	3894
											Serum globulin (g/dL)	0.0012	0.048(0.015)	3894
											(ln +1)Serum globulin: SI (g/L)	0.00087	0.015(0.0044)	3894
											Serum globulin: SI (g/L)	0.0012	0.48(0.15)	3894
											(ln +1 Serumglobulin(g/dL)	0.00092	0.011(0.0034)	3894
											Serumglobulin(g/dL)	0.0012	0.048(0.014)	3894
											(ln +1) Serumglobulin:SI(g/L)	0.00087	0.015(0.0044)	3894
											Serumglobulin:SI(g/L)	0.0012	0.48(0.15)	3894
GCKR	rs1260326	2	27730940	T	Triglycerides	Cholesterol	20657596	Vitamin A	NHW	NHANES III	Vitamin A (ug/dL)	0.0061	1.30(0.47)	2550
					lipids	Hypertriglyceridemia	22286219				Vitamin A (umol/L)	0.0061	0.045(0.017)	2550
						Metabolite levels	20139978				SerumvitaminA(ug/dL)	0.0061	1.30(0.47) 0.045(0.017)	2550
						total Hematological and biochemical traits	20081857				SerumvitaminA:SI(umol/L)	0.0061		2550
						Liver enzyme levels (gamma-glutamyl transferase)	22139419			NHANES 1999–2002	(ln +1) Vitamin A (umol/L)	0.00028	0.024 (0.0066)	1639
						C-reactive protein	20383146				Vitamin A (umol/L)	0.00012	0.082(0.021)	1639
						Waist circumference and related phenotypes	20686565				(ln +1)Vitamin A (ug/dL)	0.00045	0.035(0.0099)	1639
						Triglycerides	18454146				Vitamin A (ug/dL)	0.00011	2.34(0.61)	1639
						Chronic kidney disease	21300955			NHANES Combined	(ln +1)Vitamin A (ug/dL)	6.37E-05	0.024(0.0060)	4189
						Platelet counts	19060906				Vitamin A (ug/dL)	1.06E-05	1.65(0.37)	4189
						Two-hour glucose challenge	22001757				(ln +1)Vitamin A (umol/L)	3.49E-05	0.016(0.0040)	4189
						Cardiovascular disease risk factors	19060910				Vitamin A (umol/L)	1.08E-05	0.057(0.013)	4189
						Metabolic traits	21943158				(ln +1)SerumvitaminA(ug/dL)	6.37E-05	0.024(0.0060)	4189
											SerumvitaminA(ug/dL)	0.0000106	1.65(0.37)	4189
											(ln +1)SerumvitaminA:SI(umol/L)	3.49E-05	0.016(0.0040)	4189
											SerumvitaminA:SI(umol/L)	1.08E-05	0.057(0.013)	4189
SLC30A8	rs13266634	8	118184783	T	Type 2 Diabetes	Type 2 diabetes and other traits	17460697	Vitamin E	NHW	NHANES III	(ln +1)Vitamin E (ug/dL)	0.0086	-0.031(0.012)	2595
						Glycated hemoglobin levels	17463246				Vitamin E (ug/dL)	5.00E-03	-50.23(17.86)	2595
							19096518				Vitamin E (umol/L)	0.005	-1.16(0.41)	2595
							19734900				(ln +1)SerumvitaminE(ug/dL)	0.0086	-0.031(0.012)	2595
							17463249				SerumvitaminE(ug/dL)	0.005	-50.23(17.86)	2595
							17463248			NHANES 1999–2002	(ln +1)Vitamin E (umol/L)	0.0057	-0.041(0.015)	1627
							19056611				Vitamin E (umol/L)	0.0037	-1.70(0.58)	1627
							19401414				(ln +1)Vitamin E (ug/dL)	0.0058	-0.042(0.015)	1627
							17293870				Vitamin E (ug/dL)	0.0037	-73.28(25.17)	1627
										NHANES Combined	(ln +1)Vitamin E (ug/dL)	0.00025	-0.034(0.0095)	4222
											Vitamin E (ug/dL)	8.54E-05	-58.39(14.84)	4222
											(ln +1)Vitamin E (umol/L)	0.00024	-0.033(0.0092)	4222
											Vitamin E (umol/L)	8.54E-05	-0.034(0.0095)	4222
											(ln +1)SerumvitaminE(ug/dL)	0.00025	-58.39(14.84)	4222
											SerumvitaminE(ug/dL)	8.54E-05	-1.35(0.34)	4222
FADS1	rs174547	11	61570783	T	HDL Cholesterol	Resting heart rate	22286219	Ferritin	MA	NHANES III	(ln +1)Ferritin (ng/mL)	0.005	0.11(0.037)	1826
						HDL cholesterol	20639392				(ln +1)Ferritin (ug/L)	0.005	0.11(0.037)	1826
						Metabolic traits	21886157				(ln +1)Serumferritin(ng/mL)	0.005	0.11(0.037)	1826
						Phospholipid levels (plasma)	21829377				(ln +1)Serumferritin:SI(ug/L)	0.005	0.11(0.037)	1826
						Metabolite levels	20037589			NHANES 1999–2002	(ln +1)Ferritin (ug/L)	0.0089	0.10(0.040)	1861
						Triglycerides	19060906				(ln +1)Ferritin (ng/mL)	0.0089	0.10(0.040)	1861
						Lipid metabolism phenotype				NHANES Combined	Ferritin (ng/mL)	0.0064	9.16(3.35)	3687
											Ferritin (ug/L)	0.0064	9.16(3.35)	3687
											Serumferritin(ng/mL)	0.0064	9.16(3.35)	3687
											Serumferritin:SI(ug/L)	0.0064	9.16(3.35)	3687
FADS1	rs174547	11	61570783	T	HDL Cholesterol	Resting heart rate	20639392	Folate	NHB	NHANES III	(ln +1)RBC folate (ng/mL)	0.0013	-0.079(0.025)	2028
						HDL cholesterol	21886157				RBC folate (ng/mL)	0.00015	-15.14(3.98)	2028
						Metabolic traits	21829377				(ln +1)RBC folate: SI (nmol/L)	0.0013	-0.080(0.025)	2028
						Phospholipid levels (plasma)	20037589				RBC folate: SI (nmol/L)	0.00015	-34.31(9.029)	2028
						Metabolite levels	19060906				(ln +1)RBCfolate(ng/mL)	0.0013	-0.079(0.025)	2028
						Triglycerides	22286219				RBCfolate(ng/mL)	0.00015	-15.14(3.98)	2028
						Lipid metabolism phenotypes					(ln +1)RBCfolate:SI(nmol/L)	0.0013	-0.080(0.025)	2028
											RBCfolate:SI(nmol/L)	0.00015	-34.31(9.029)	2028
										NHANES 1999–2002	Folate, serum (nmol/L)	0.0013	-4.54(1.41)	1330
											Folate, RBC (nmol/L RBC)	0.002	-56.51(18.25)	1329
											Folate, serum (ng/mL)	0.0013	-2.0036(0.62)	1330
											Folate, RBC (ng/mL RBC)	0.002	-24.95 (8.059)	1329
										NHANES Combined	Serum folate (ng/mL)	0.0087	-0.82(0.31)	3366
											Serum folate: SI (nmol/L)	0.0087	-1.85(0.70)	3366
											RBC folate (ng/mL)	0.00039	-15.81(4.45)	3357
											RBC folate: SI (nmol/L)	0.00039	-35.82(10.080)	3357
											RBCfolate(ng/mL)	0.00039	-15.81(4.45)	3357
											RBCfolate:SI(nmol/L)	0.00039	-35.82(10.080)	3357
											Serumfolate(ng/mL)	0.0087	-0.82(0.31)	3366
											Serumfolate:SI(nmol/L)	0.0087	-1.85(0.70)	3366
LIPC	rs1800588	15	58723675	T	HDL Cholesterol	HDL cholesterol	18193044	Folate	MA	NHANES III	Serum folate (ng/mL)	0.0089	-0.32(0.12)	1996
											Serum folate: SI (nmol/L)	0.0088	-0.73(0.28)	1996
											Serumfolate(ng/mL)	0.0089	-0.32(0.12)	1996
											Serumfolate:SI(nmol/L)	0.0088	-0.73(0.28)	1996
										NHANES 1999–2002	(ln +1)Folate, RBC (nmol/L RBC)	0.0001	-0.047(0.012)	1838
											Folate, RBC (nmol/L RBC)	0.00054	-31.060(8.96)	1838
											(ln +1)Folate, RBC (ng/mL RBC)	0.0001	-0.047(0.012)	1838
											Folate, RBC (ng/mL RBC)	0.00054	-13.71(3.95)	1838
										NHANES Combined	(ln +1)Serum folate (ng/mL)	0.0074	-0.035(0.013)	3837
											Serum folate (ng/mL)	0.003	-0.46(0.15)	3837
											(ln +1)Serum folate: SI (nmol/L)	0.0083	-0.037(0.014)	3837
											Serum folate: SI (nmol/L)	0.003	-1.058(0.35)	3837
											(ln +1)RBC folate (ng/mL),	0.002	-0.032(0.010)	3815
											RBC folate (ng/mL)	0.00045	-9.38(2.67)	3815
											(ln +1)RBC folate: SI (nmol/L)	0.002	-0.032(0.010)	3815
											RBC folate: SI (nmol/L)	0.00045	-21.26(6.053)	3815
											(ln +1)RBCfolate(ng/mL)	0.002	-0.032(0.010)	3815
											RBCfolate(ng/mL)	0.00045	-9.38(2.67)	3815
											(ln +1)RBCfolate:SI(nmol/L)	0.002	-0.032(0.010)	3815
											RBCfolate:SI(nmol/L)	0.00045	-21.26(6.053)	3815
											(ln +1) Serumfolate(ng/mL)	0.0074	-0.035(0.013)	3837
											Serumfolate(ng/mL)	0.003	-0.46(0.15)	3837
											(ln +1)Serumfolate:SI(nmol/L)	0.0083	-0.037(0.014)	3837
											Serumfolate:SI(nmol/L)	0.003	-1.058(0.35)	3837
LIPC	rs1800588	15	58723675	T	HDL Cholesterol	HDL cholesterol	18193044	Vitamin E	NHW	NHANES III	(ln +1)Vitamin E (ug/dL)	0.00059	0.044(0.013)	2553
											Vitamin E (ug/dL)	0.0035	56.21(19.26)	2553
											Vitamin E (umol/L)	0.0035	1.31(0.45)	2553
											(ln +1)SerumvitaminE(ug/dL)	0.00059	0.044(0.013)	2553
											SerumvitaminE(ug/dL)	0.0035	56.21(19.26)	2553
										NHANES 1999–2002	(ln +1)Vitamin E (umol/L)	0.00065	0.060(0.017)	1609
											Vitamin E (umol/L)	0.0017	2.15(0.69)	1609
											(ln +1)Vitamin E (ug/dL)	0.00063	0.062(0.018)	1609
											Vitamin E (ug/dL)	0.0017	92.75(29.57)	1609
										NHANES Combined	(ln +1)Vitamin E (ug/dL)	2.32E-05	0.045(0.010)	4162
											Vitamin E (ug/dL)	0.0002	61.83(16.58)	4162
											(ln +1)Vitamin E (umol/L)	2.37E-05	0.043(0.010)	4162
											Vitamin E (umol/L)	0.0002	1.43(0.38)	4162
											(ln +1)SerumvitaminE(ug/dL)	2.32E-05	0.045(0.010)	4162
											SerumvitaminE(ug/dL)	0.0002	61.83(16.58)	4162
ABCG2	rs2231142	4	89052323	C	Gout	Uric acid levels	22229870	Blood Pressure (Diastolic)	MA	NHANES III	(ln +1)K5 for second BP measure(diastolic, mmHg)	0.0003	0.044(0.012)	1697
							18834626				K5 for second BP measure(diastolic, mmHg)	0.00098	1.76(0.53)	1697
							19503597				(ln +1)K5 for third BP measure (diastolic, mmHg)	0.0063	0.032(0.012)	1694
											K5 for third BP measure (diastolic, mmHg)	0.0046	1.51(0.53)	1694
											(ln +1)Overall average K5, diastolic BP(age5+)	1.45E-06	0.033(0.0068)	2023
											Overall average K5, diastolic, BP(age5+)	2.21E-06	2.19(0.46)	2023
										NHANES 1999–2002	BPXDI3:Diastolic: Blood pres(3rd rdg) mm Hg	0.0045	1.69(0.59)	1605
										NHANES Combined	K5 for first BP measure (diastolic, mmHg)	0.0088	1.092(0.41)	3338
											(ln +1) K5 for second BP measure(diastolic, mmHg)	0.0033	0.032(0.011)	3340
											K5 for second BP measure(diastolic, mmHg)	2.89E-05	1.66(0.39)	3340
											K5 for third BP measure (diastolic, mmHg)	3.98E-05	1.65(0.40)	3299
											Overall average K5, diastolic, BP(age5+)	5.21E-07	1.82(0.36)	3837
ABCG2	rs2231142	4	89052323	C	Gout	Urate levels	22229870	Protoporphyrin	MA	NHANES III	(ln +1)Protoporphyrin (ug/dL RBC)	2.61E-07	-0.075(0.015)	2029
							19503597				protoporphyrin (ug/dL RBC)	0.004	-3.92(1.36)	2029
							18834626				(ln +1)Protoporphyrin (umol/L RBC)	7.87E-06	-0.037(0.0083)	2029
											Protoporphyrin (umol/L RBC)	0.004	-0.070(0.024)	2029
										NHANES 1999–2002	(ln +1)Protoporphyrin (umol/L RBC)	0.00037	-0.042(0.012)	968
											Protoporphyrin (umol/L RBC)	0.0018	-0.094(0.030)	968
											(ln +1)Protoporphyrin (ug/dL RBC)	0.0002	-0.079(0.021)	968
											Protoporphyrin (ug/dL RBC)	0.0018	-5.321(1.70)	968
										NHANES Combined	Protoporphyrin (ug/dL RBC)	9.41E-08	5.21(0.97)	3897
											Protoporphyrin (umol/L RBC)	9.85E-08	0.092(0.017)	3897
ABCG2	rs2231142	4	89052323	C	Gout	Urate levels	22229870	Protoporphyrin	NHW	NHANES III	(ln+1)Protoporphyrin (ug/dL RBC)	6.00E-06	-0.062(0.014)	2587
							19503597				(ln+1)Protoporphyrin (ug/dL RBC)	6.00E-06	-0.062(0.014)	2587
							18834626				(ln+1)Protoporphyrin (umol/L RBC)	9.60E-06	-0.032(0.0073)	2587
											(ln+1)Protoporphyrin (umol/L RBC)	9.60E-06	-0.032(0.0073)	2587
											Protoporphyrin (umol/L RBC)	3.70E-05	-0.087(0.021)	2587
											Protoporphyrin (umol/L RBC)	3.60E-05	-0.087(0.021)	2587
											Protoporphyrin (ug/dL RBC)	3.76E-05	-4.88(1.18)	2587
											Protoporphyrin (ug/dL RBC)	3.76E-05	-4.88(1.18)	2587
										NHANES 9902	(ln+1)Protoporphyrin (ug/dL RBC)	6.60E-04	-0.060(0.01)	1667
											(ln+1)Protoporphyrin (umol/L RBC)	0.0012	-0.029(0.0092)	1667
											Protoporphyrin (umol/L RBC)	0.0064	-0.058(0.021)	1667
											Protoporphyrin (ug/dL RBC)	0.0065	-3.28(1.20)	1667
KCTD10	rs2338104	12	109895168	G	HDL Cholesterol	HDL cholesterol	19060906	Hearing	NHW	NHANES III	Right Threshold @ 1000Hz-Second Reading	0.0034	2.20(0.74)	258
							18193043				Rightearairhearlvl, repeat, 1000Hz(dB)	0.0034	2.20(0.74)	258
										NHANES 1999–2002	Right threshold @ 500Hz	0.006	1.11(0.40)	1415
										NHANES Combined	Right threshold @ 1000Hz	0.0071	1.010(0.37)	1669
											Right Threshold @ 1000Hz-Second Reading	0.0028	1.14(0.381)	1673
											Right threshold @ 500Hz	0.0023	1.12(0.36)	1673
											Rightearairhearlevel, first, 1000Hz(dB	0.0071	1.010(0.37)	1669
											Rightearairhearlvl, repeat,1000Hz(dB)	0.0028	1.14(0.38)	1673
											Rightearairhearinglevel, 500Hz(dB)	0.0023	1.12(0.36)	1673
KCTD10	rs2338104	12	109895168	G	HDL Cholesterol	HDL cholesterol	19060906	Hemoglobin	NHW	NHANES III	(ln +1)Mean cell hemoglobin: SI (pg)	0.0016	-0.0050(0.0016)	2582
							18193043				Mean cell hemoglobin: SI (pg)	0.0017	-0.15 (0.048)	2582
											(ln +1)Meancellhemoglobin:SI(pg)	0.0016	-0.0050 (0.0016)	2582
											Meancellhemoglobin:SI(pg)	0.0017	-0.15 (0.048)	2582
										NHANES 1999–2002	(ln +1)Mean Cell Hemoglobin Concentration (MCHC) (g/dL)	0.0024	-0.0015(0.00048)	3964
											Mean Cell Hemoglobin Concentration (MCHC) (g/dL)	0.0025	-0.050 (0.017)	3964
											(ln +1)Mean cell hemoglobin (pg)	0.0018	-0.0042(0.0013)	3964
											Mean cell hemoglobin (pg)	0.0028	-0.12(0.042)	3964
										NHANES Combined	(ln +1) Mean cell hemoglobin: SI (pg)	2.16E-05	-0.0044(0.0010)	6546
											Mean cell hemoglobin: SI (pg)	3.85E-05	-0.13(0.031)	6546
											(ln +1)Mean cell hemoglobin concentration	0.0038	-0.001(0.00036)	6546
											Mean cell hemoglobin concentration	0.0043	-0.036(0.012)	6546
											(ln +1) Meancellhemoglobin:SI(pg)	2.16E-05	-0.0044(0.0010)	6546
											Meancellhemoglobin:SI(pg)	3.85E-05	-0.13(0.031)	6546
											(ln +1)Meancellhemoglobinconcentration	3.80E-03	-0.001(0.00036)	6546
											Meancellhemoglobinconcentration	0.0043	-0.036(0.012)	6546
BUD13	rs28927680	11	116619073	G	HDL Cholesterol	Triglycerides	18193044	Vitamin E	NHW	NHANES III	(ln +1)Vitamin E (ug/dL)	4.45E-05	-0.086 (0.021)	2596
											Vitamin E (ug/dL)	0.00063	-109.024 (31.84)	2596
											Vitamin E (umol/L)	0.00063	-2.53 (0.74)	2596
											(ln +1)SerumvitaminE(ug/dL)	4.45E-05	-0.086 (0.021)	2596
											SerumvitaminE(ug/dL)	0.00063	-109.024 (31.84)	2596
										NHANES 1999–2002	(ln +1)Vitamin E (umol/L)	0.001	-0.090 (0.027)	1624
											Vitamin E (umol/L)	0.0033	-3.17(1.077)	1624
											(ln +1)Vitamin E (ug/dL)	0.001	-0.093 (0.028)	1624
											Vitamin E (ug/dL)	0.0033	-136.39(46.39)	1624
										NHANES Combined	(ln +1)Vitamin E (ug/dL)	1.34E-07	-0.090(0.017)	4220
											Vitamin E (ug/dL)	5.36E-06	-122.098(26.79)	4220
											(ln +1)Vitamin E (umol/L)	1.40E-07	-0.087(0.016)	4220
											Vitamin E (umol/L)	5.36E-06	-2.83(0.62)	4220
											(ln +1)SerumvitaminE(ug/dL)	1.34E-07	-0.090(0.017)	4220
											SerumvitaminE(ug/dL)	5.36E-06	-122.098(26.79)	4220
RPS26P35 - TNFRSF11B	rs4355801	8	119923873	G	Bone mineral density	Bone mineral density	18455228	White Blood Cell	NHB	NHANES III	White blood cell count: SI	0.0036	0.30 (0.10)	2077
											Whitebloodcellcount	3.60E-03	0.30(0.10)	2077
											Whitebloodcellcount:SI	0.0036	0.30(0.10)	2077
										NHANES 1999–2002	White blood cell count SI	0.0079	0.378(0.14)	1334
										NHANES Combined	(ln +1)White blood cell count: SI	5.77e-05 7.19e-05 5.77e-05 7.19e-05	0.042(0.010),	3411
											White blood cell count: SI		0.33(0.083),	3411
											(ln +1)Whitebloodcellcount:SI		0.042(0.010),	3411
											Whitebloodcellcount:SI		0.33(0.083)	3411
SLC2A9	rs6855911	4	9935910	G	Uric acid	Urate levels	17997608	Body Measurements (Leg)	NHB	NHANES III	Thigh circumference (cm)(2 yrs and over)	0.0012	0.80(0.25)	1972
										NHANES 1999–2002	(ln +1)Thigh Circumference (cm)	0.0087	0.014 (0.0055)	1256
											Thigh Circumference (cm)	0.0061	0.89 (0.33)	1256
										NHANES Combined	(ln +1)Thigh circumference (cm)(2 yrs and over)	1.09E-05	0.015(0.0034)	3228
											Thigh circumference (cm)(2 yrs and over)	6.12E-06	0.90(0.19)	3228
APOB - KLHL29	rs562338	2	21288321	T	LDL Cholesterol	LDL Cholesterol	1.83E+15	Hearing	NHB	NHANES III	(Ln+1)Leftearairhearlvl, repeat,1000Hz(dB	0.0071	0.20(0.075)	377
										NHANES 9902	Left threshold @ 1000Hz (db)	0.0096	1.99(0.77)	528
GCKR	rs780094	2	27741237	G	Metabolic syndrome	C-reactive Protein	22581228	Potassium intake	MA	NHANES III	Potassium(mg)	0.0043	-132.98(0.66)	1961
					LDL cholesterol	glucose/HOMA-B	22399527				(ln+1)Potassium(mg)	0.0053	-0.050(0.66)	1961
					Uric acid levels	T2D	21886157							
					Metabolic traits		21829377							
					C-reactive protein		20081858							
					Phospholipid levels (plasma)		19503597							
					Triglycerides		18439548							
					Fasting glucose-related traits		18193044							
					Fasting insulin-related traits		18193043							
							18179892							
										NHANES 9902	Potassium (mg)	0.0047	-139.47(0.67)	1798
GCKR	rs780094	2	27741237	G	Metabolic syndrome	C-reactive Protein	22581228	Vitamin B6 intake	MA	NHANES III	VitaminB6(mg)	0.0098	-0.10(0.66)	1961
					LDL cholesterol	glucose/HOMA-B	22399527			NHANES 9902	Vitamin B6 (mg)	0.0042	-0.11274(0.67)	1798
					Uric acid levels	T2D	21886157							
					Metabolic traits		21829377							
					C-reactive protein		20081858							
					Phospholipid levels (plasma)		19503597							
					Triglycerides		18439548							
					Fasting glucose-related traits		18193044							
					Fasting insulin-related traits		18193043							
							18179892							
None	rs1800795	7	22766645	G	C-reactive Protein	N/A	15820616	White Blood Cell Count	NHB	NHANES III	White blood cell count: SI	0.0047	-0.34(0.12)	2038
							19435922			NHANES 9902	(ln+1)Segmented Neutrophils number	0.0048	-0.071(0.025)	1316
							19452524				(ln+1)White blood cell count SI	0.0071	-0.054(0.020)	1324
							19140096				Segmented Neutrophils number	0.0083	-0.325(0.12)	1316
							19267250			NHANES Combined	(ln+1)White blood cell count: SI	7.01E-05	-0.047(0.011)	3362
							19280716				White blood cell count: SI	1.60E-04	-0.36(0.096)	3362
							19330901							
							19377912							
							19387461							
							20149313							
							20175976							
							20176930							
							20333461							
							20361391							
							20436380							
							20459474							
							20592333							
							20622166							
							19542902							
							19592000							
							19671870							
							19833146							
							19853505							
							19853505							
							19876004							
							20043205							
							20132806							
							16544245							
							16644865							
							17003362							
							17416766							
							17623760							
							17694420							
							17916900							
							17996468							
							18041006							
							18239642							
							18257935							
							18276608							
							18321738							
							18449426							
							18458677							
							18752089							
							18992263							
							19056105							
							19106168							
							20044998							
KCNQ1	rs2237895	11	2857194	C	Type 2 Diabetes	Type 2 Diabetes	20174558	Body Measurement (Arm)	MA	NHANES III	Arm circumference(cm)(2 months and over)	0.0019	-0.47(0.15)	1987
											(ln+1)Arm circumference(cm)(2 months and over)	0.002	-0.015(0.0047)	1987
										NHANES 9902	(ln+1)Upper Arm Length (cm)	0.0061	-0.0062(0.0022)	1835
											Upper Arm Length (cm)	0.0068	-0.23(0.084)	1835
None	rs1529729	19	11163562	G	LDL-C	None	18714375	Broken/Fractured Bone	NHW	NHANES III	Doctor told had broken/fractured spine	0.0042	0.493(0.25)	2303
										NHANES 9902	(ln+1)Broken or fractured spine	0.002	1.54(0.14)	3933
											Broken or fractured spine	0.002	1.54(0.14)	3933

Novel PheWAS results with the same SNP, phenotype class, and race-ethnicity across NHANES, ordered by these variables. Information on the closest gene and previously reported phenotypes from the PAGE network and GWAS Catalog (with PubMed ID) are included for each SNP. Long phenotype description, along with the corresponding p-value, beta (SE  =  standard error), coded allele frequency (CAF), and sample size for the association are also listed. Significant measures from NHANES III and Continuous NHANES are included, and NHANES Combined was also included when the phenotype was harmonized across both surveys.

Phenotypes correlated with r>0.6 with any of the significant traits, in either NHANES III or Continuous NHANES for the race/ethnicity of significant PheWAS result.

### Replication of Known Results

**Figure 2:**
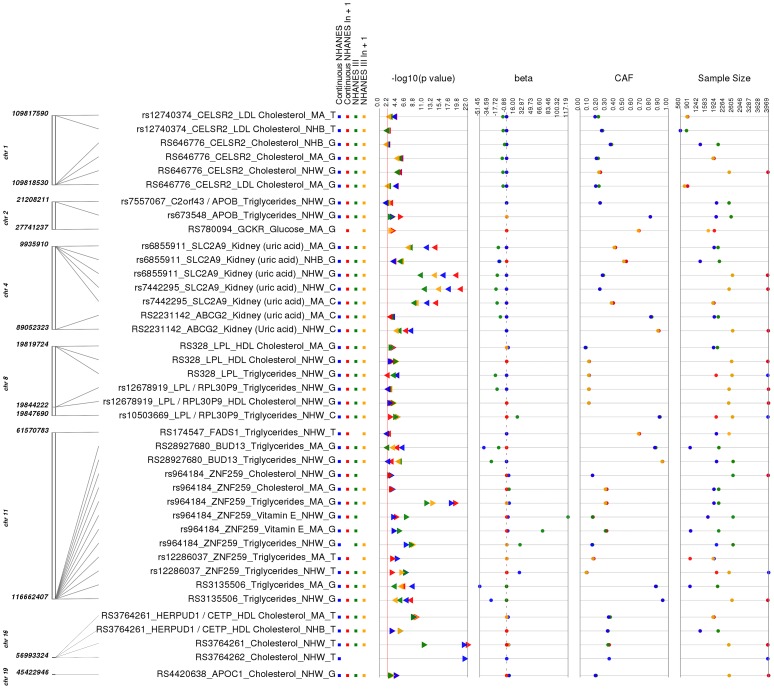
Replicating results for PheWAS. This is a plot of SNP-phenotype associations observed in both NHANES III and Continuous NHANES with p-value <0.01, for SNPs with an allele frequency >0.01, and a sample size >200, for the same race-ethnicity, phenotype-class, and direction of effect. Plotted are results where the significant SNP-phenotype association matches a previously reported SNP-Phenotype association. The first column indicates the chromosome and base pair location of the SNP. The second column indicates the SNP ID, the associated phenotype-class, the self-reported race-ethnicity (NHW  =  Non-Hispanic Whites, NHB  =  Non-Hispanic Blacks, or MA  =  Mexican Americans), and the coded-allele. The next column contains a colored box if association results were available for natural log transformed Continuous NHANES (Continuous NHANES ln+1), un-transformed Continuous NHANES phenotypes, NHANES III untransformed phenotypes (NHANES III), or transformed NHANES III phenotypes (NHANES III ln+1) (see methods for more details on phenotype transformation). The next column indicates the p-value for each association, and the triangle direction indicates whether the association had a positive (triangle pointed to the left) or negative direction of effect (triangle pointed to the right). The following columns indicate magnitude of the effect (beta), the coded allele frequency (CAF), and the sample size for the association.

As a positive control, we first sought evidence for associations that replicate findings from the literature. Replication of previously reported associations validates our PheWAS pipeline and data integrity. Thirty-nine out of the 69 (56.5%) of our PheWAS associations have previously been described in the literature with the same direction of effect, and our results for these associations are presented in S2 and S3 Tables as well as visualized in Fig. 2. A proportion of the phenotypes could have phenotypic harmonization such that we could explore the association result for the phenotype across both surveys, NHANES III and Continuous NHANES, which we refer to as *NHANES Combined*. A Combined NHANES result was not available for every phenotype, as not all phenotypes could be harmonized across both surveys even if phenotypes could be binned into phenotype classes across both surveys. Our result tables contain this NHANES Combined information when available.

The majority of the SNPs within our study (51 out of 80), but not all of the SNPs, were chosen for genotyping based on published lipid trait genetic association studies (for example, [10]–[12]), and of these, 19/23 lipid-associated SNPs were associated with lipid traits in this PheWAS. For example, total cholesterol levels and LDL cholesterol levels have been previously associated with the SNP rs646776 near *CELSR2* in European-descent populations [13]–[15]. In this PheWAS, we observed a significant association between rs646776 (coded allele G) and total cholesterol levels in NHANES III (p = 3.17×10^−6^, β = −7.66, n = 2,224) and Continuous NHANES (p = 9.15×10^−7^, β = −0.014, n = 3,943) for non-Hispanic whites with the same direction of effect as the association previously reported for this SNP and LDL cholesterol levels. The association between rs646776 and total cholesterol remained significant in Combined NHANES (p = 1.0×10^−10^, β = −0.029, n = 6,389).

### Related Associations

**Figure 3:**
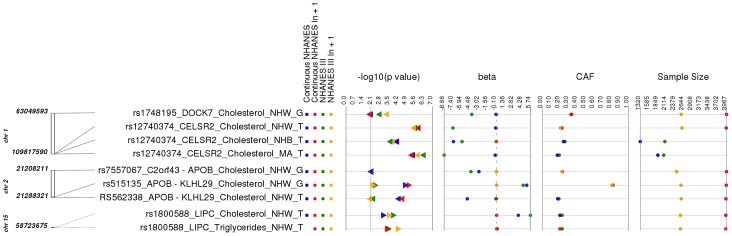
Related results for PheWAS. This is a plot of SNP-phenotype associations observed in both NHANES III and Continuous NHANES with p-value <0.01, for SNPs with an allele frequency >0.01, and a sample size >200, for the same race-ethnicity, phenotype-class, and direction of effect. Plotted are results where the significant SNP-phenotype association is closely related to the phenotype of a previously reported SNP-Phenotype association. The first column indicates the chromosome and bp location of the SNP. The second column indicates the SNP ID, the associated phenotype-class, the self-reported race-ethnicity (NHW  =  Non-Hispanic Whites, NHB  =  Non-Hispanic Blacks, or MA  =  Mexican Americans), and the coded-allele. The next column contains a colored box if association results were available for natural log transformed Continuous NHANES phenotypes (Continuous NHANES ln+1), un-transformed Continuous NHANES phenotypes, NHANES III untransformed phenotypes (NHANES III), or transformed NHANES III phenotypes (NHANES III ln+1) (see methods for more details on phenotype transformation). The next column indicates the p-value for each association, and the triangle direction indicates whether the association had a positive (triangle pointed to the left) or negative direction of effect (triangle pointed to the right). The following columns indicate magnitude of the effect (beta), the coded allele frequency (CAF), and the sample size for the association.

After determining results where the phenotype of our association matched that of the same SNP-phenotype association in the GWA catalog, we evaluated whether any of our phenotypes were extremely similar to previously published SNP-phenotype associations. There were a total of 9/69 (∼13%) PheWAS results where the SNPs had been previously associated with lipid measurements not exactly matching the respective lipid measurements of our study (S4 Table and Fig. 3). For example, the SNP rs515135 near *APOB/KLHL29* has been previously reported to be associated with LDL cholesterol (LDL-C) levels in European-descent populations [16], [17]. In this PheWAS, rs515135 (coded allele G) was associated with total cholesterol levels in non-Hispanic whites. For this SNP, the most significant results meeting our PheWAS replication criteria from NHANES III were: p = 0.0024, β = 4.85, n = 2,569 and Continuous NHANES were: p = 1.06×10^−5^, β = 0.026, n = 3959. This variant was also associated with total cholesterol levels in Combined NHANES (p = 1.39×10^−7^, β = 5.13, n = 6,528).

Another example of a closely related association was for SNP rs7557067 near *APOB*, previously found to be associated with triglyceride levels in European-descent populations [17]. In this PheWAS, rs7557067 (coded allele G) was associated with total cholesterol levels in non-Hispanic whites from NHANES III (p = 0.0050, β = −0.012, n = 2,436) and Continuous NHANES (p = 0.0053, β = −0.015, n = 3,966). In the larger sample size of Combined NHANES, this association with total cholesterol levels was maintained (p = 1.1×10^−4^, β = −0.014, n = 6,404). Given that total cholesterol includes HDL-C and that HDL-C is inversely correlated with triglycerides [18], [19], this PheWAS finding was also expected.

### Novel Associations

The remainder of the PheWAS results with phenotypes that did not match previously reported SNP-phenotype associations had phenotypes very distinct from previously reported phenotypes. A total of 21/69 (∼30%) PheWAS results are potentially novel findings. These are associations with a greater divergence between the previously associated phenotype for a given SNP and the associated phenotype found in this study (Table 3). We found novel results for all three racial/ethnic groups. However, only one novel result meeting our PheWAS significance criteria generalized across two or more populations showing the same direction of effect: protoporphyrin levels in both non-Hispanic whites and Mexican Americans for the *ABCG2* SNP rs2231142 (coded allele C). Of the replicating measures for protoporphyrin levels, the most significant results for this association in Mexican Americans for NHANES III was: p = 2.61×10^−7^, β = −0.075, n = 2,029, for Continuous NHANES was: p = 2.0×10^−4^, β = −0.079, n = 968, and for Combined NHANES: p = 9.41×10^−8^, β = −5.21, n = 3,897. The most significant result for this association in non-Hispanic whites was for NHANES III: p = 6.0×10^−6^, β = −0.062, n = 2,587 and for Continuous NHANES was: p = 6.6×10^−4^, β = −0.06, n = 1,667. This SNP was previously associated with uric acid [20]–[23]. We also found this SNP to be associated with uric acid in non-Hispanic whites and Mexican Americans with the same direction of effect as previously reported associations, as well as an additional novel result for blood pressure measurements only in Mexican Americans with an opposite direction of effect. The number of novel results was similar across race-ethnicities, even with the difference in sample size across non-Hispanic whites, non-Hispanic blacks, and Mexican Americans that could affect power for detection of novel associations.

An example novel result showing a very unique divergence from previously reported associations was for the SNP rs11206510 (coded allele T) near the gene *PCSK9*. This SNP has been previously associated with coronary heart disease [24], LDL-C [16], [17], [25], and myocardial infarction [26] in European-descent populations, but we did not replicate any of those previously reported associations. In this study we found this SNP was associated with serum globulin levels in Mexican Americans from NHANES III (p = 0.0095, β = 0.0120, n = 2,023), Continuous NHANES (p = 0.0042, β = 0.012, n = 1871), and Combined NHANES (p = 8.7×10^−4^, β = 0.015, n = 3,894). We contrasted the direction of effect of this SNP with the previously reported associations for this SNP and the direction of effect was the same.

Another example of novel divergence from previously reported results involved two SNPs we found to be associated with white blood cell count in non-Hispanic blacks. The SNP rs1800795 (coded allele G) near *IL6* previously was associated with C-reactive protein levels [27]–[29]. In our study, this SNP was associated with white blood cell counts in non-Hispanic blacks from NHANES III (p = 0.0047, β = −0.34, n = 2038) and Continuous NHANES (p = 0.0048, β = −0.071, n = 1,316). We also found that rs4355801 in *TNFRSF11B* was associated with white blood cell counts in non-Hispanic blacks from NHANES III (p = 0.0036, β = 0.30, n = 6,991), Continuous NHANES (p = 0.0079, β = 0.378, n = 3,728), and Combined NHANES (p = 5.77×10^−5^, β = 0.042, n = 3,411). Previously, *TNFRSF11B* rs4355801 (coded allele G) was associated with bone mineral density in women of European-descent [30]. We did not observe a significant PheWAS association with C-reactive protein or bone mineral density in our study for these two SNPs, respectively.

We found a total of six novel PheWAS-significant results associated with circulating vitamin levels (vitamin E, vitamin A, and folate). For example, a PheWAS-significant association for the missense SNP rs1260326 (coded allele T) in the gene *GCKR* was found with vitamin A levels in non-Hispanic whites from NHANES III (p = 6.1×10^−3^, β = 1.30, n = 2,250), Continuous NHANES (p = 1.11×10^−4^, β = 2.34, n = 1,639), and Combined NHANES (p = 1.06×10^−5^, β = 1.65, n = 4,189). This SNP was previously associated with serum albumin levels and serum total protein levels in European- and Japanese-descent individuals [31], non-albumin protein levels in Japanese-descent individuals [32], platelet counts [33], cardiovascular disease risk factors [34], C-reactive protein levels [35], urate levels [20], total cholesterol and triglyceride levels [36], and chronic kidney disease [37] in individuals of European ancestry, and liver enzyme levels in European- and Asian-descent populations [38]. None of these previously reported associations replicated in our study. We compared the positive direction of effect of this SNP rs1260326, associated with vitamin levels, with previously reported associations. Associations with the same coded allele (T) with urate levels [20], serum albumin levels [31], serum total protein levels [31], platelet counts [33], liver enzyme levels[38], cardiovascular disease risk factors [34], C-reactive protein levels [35], total cholesterol and triglyceride levels [36], chronic kidney disease [37] all had a positive direction of effect. This SNP was associated with non-albumin protein levels [32] with a negative direction of effect.

### Identification of Pleiotropy

**Figure 4:**
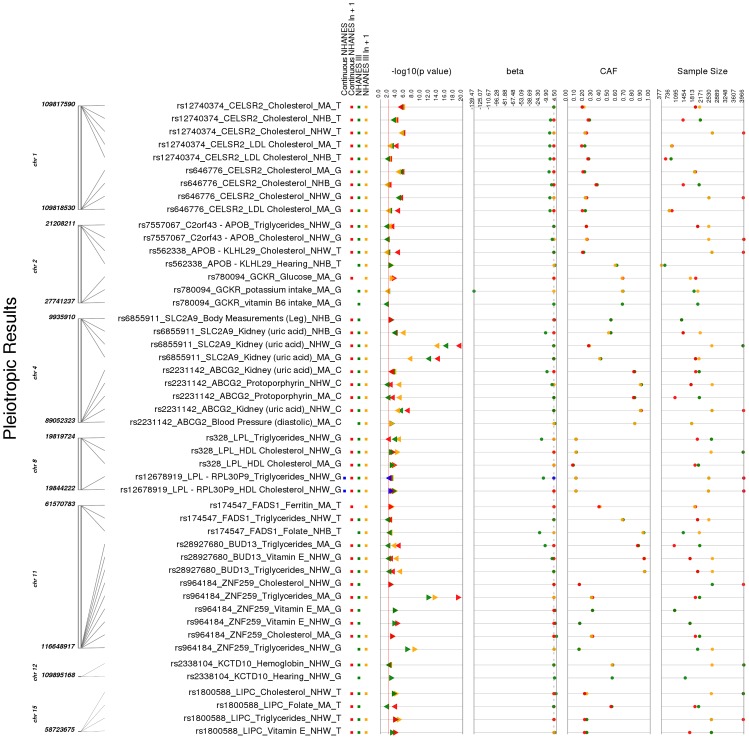
Potentially pleiotropic results. These are the PheWAS-significant results of this study with more than one distinct phenotype-class associated with the same SNP. This is a plot of SNP-phenotype associations observed in both NHANES III and Continuous NHANES with p-value <0.01, for SNPs with an allele frequency >0.01, and a sample size >200, for the same race-ethnicity, phenotype-class, and direction of effect. Plotted are results where the significant SNP-phenotype association matches a previously reported SNP-Phenotype association. The first column indicates the chromosome and bp location of the SNP. The second column indicates the SNP ID, the associated phenotype-class, the self-reported race-ethnicity (NHW  =  Non-Hispanic Whites, NHB  =  Non-Hispanic Blacks, or MA  =  Mexican Americans), and the coded-allele. The next column contains a colored box if association results were available for natural log transformed NHANES III phenotypes (NHANES III ln+1), un-transformed NHANES III phenotypes (NHANES III), or natural log transformed Continuous NHANES phenotypes (Continuous NHANES ln+1) (see methods for more details on phenotype transformation), or untransformed Continuous NHANES phenotypes. The next column indicates the p-value for each association, and the triangle direction indicates whether the association had a positive (triangle pointed to the left) or negative direction of effect (triangle pointed to the right). The following columns indicate magnitude of the effect (beta), the coded allele frequency (CAF), and the sample size for the association.

While any of the novel PheWAS associations indicate potential pleiotropy as all of the SNPs of this study have previously reported genome-wide associations, within our study, we found 13 SNPs with more than one significant PheWAS phenotype class (Table 4 and Fig. 4). While the majority of these were SNPs were associated with more than one lipid phenotype, there were nine SNPs associated with other phenotypes.

**Table 4:** Pleiotropic results.

Nearest Gene	Distance to Gene	SNP	Coded Allele	Regulatory Evidence	Context	Previously Published Phenotype (NHGRI GWAS Catalog)	What Gene (BF)	Phenotype-Classes
*LPL*	19452	rs12678919	G	no	Intergenic	HDL Cholesterol, Triglycerides	N/A	HDL-Cholesterol (NHW +), Triglycerides (NHW +)
*APOB*	21376	rs562338	T	no	Intergenic	LDL Cholesterol	N/A	Cholesterol (NHW -), Hearing (NHB +)
*FADS1*	0	rs174547	T	6: Minimal binding evidence, motif hit	intron	Resting heart rate, HDL cholesterol, Metabolic traits, Phospholipid levels (plasma), Metabolite levels, Triglycerides, Lipid metabolism phenotypes	FADS1	Ferritin (MA +), Folate (NHB -), Triglycerides (NHW -)
*KCTD10*	0	rs2338104	G	6: Minimal binding evidence, motif hit	intron	HDL Cholesterol	KCTD10	Hearing (NHW +), Hemoglobin (NHW -)
*GCKR*	0	rs780094	G	3a: Less likely to affect binding, TF binding + any motif + DNase peak	intron	Metabolic syndrome, Phospholipid levels (plasma), Triglycerides, Fasting glucose-related traits, LDL cholesterol, Fasting insulin-related traits, Uric acid levels, Metabolic traits, C-reactive protein	GCKR	Glucose (MA +), Potassium intake (MA -), Vitamin B6 intake (MA -)
*SLC2A9*	0	rs6855911	G	no	intron	Urate Levels	SLC2A9	Kidney (Uric Acid) (MA, NHB, -), Body measurements (NHB +)
*CELSR2*	152	rs646776	G	1f: Likely to affect binding and linked to expression of a gene target, eQTL + TF binding/DNase peak	nearGene-3	Coronary heart disease, Response to statin therapy, Cholesterol, total, Progranulin levels, Myocardial infarction (early onset), LDL cholesterol	N/A	Cholesterol (MA, NHB, NHW -), LDL Cholesterol (MA -)
*ZNF259*	359	rs964184	G	1f: Likely to affect binding and linked to expression of a gene target, eQTL + TF binding/DNase peak	nearGene-3	Cholesterol, total, LDL cholesterol, Phospholipid levels (plasma), Hypertriglyceridemia, Vitamin E levels, Metabolic syndrome, Triglycerides, Lipoprotein-associated phospholipase A2 activity and mass, HDL cholesterol, Coronary heart disease	N/A	Cholesterol (MA, NHW +), Triglycerides (MA, NHW +), Vitamin E (MA, NHW, +)
*LIPC*	500	rs1800588	T	4: Minimal binding evidence, TF binding + DNase peak	nearGene-5	HDL cholesterol	N/A	Cholesterol (NHW +), Folate (MA -), Triglycerides (NHW +), Vitamin E (NHW +)
*ABCG2*	0	rs2231142	C	5: Minimal binding evidence, TF binding or DNase Peak	STOP-GAIN	Uric acid levels, Urate levels	ABCG2	Blood Pressure (diastolic) (MA +), Kidney (Uric Acid) (MA, NHW -), Protoporphyrin (MA, NHW -)
*LPL*	0	rs328	G	5: Minimal binding evidence, TF binding or DNase Peak	STOP-GAIN	HDL cholesterol, Triglycerides	LPL	HDL Cholesterol (MA, NHW +), Triglycerides (NHW -)
*CELSR2*	0	rs12740374	T	4: Minimal binding evidence, TF binding + DNase peak	UTR-3	LDL cholesterol, Coronary heart disease	CELSR2	Cholesterol (MA, NHB, NHW -), LDL Cholesterol (MA, NHB -)
*BUD13*	0	rs28927680	G	no	UTR-3	Triglycerides	BUD13	Triglycerides (MA, NHW -), Vitamin E (NHW -)

These are the SNPs of this study that had a PheWAS significant association with more than one phenotype. Results marked with a star in the phenotype-class column indicate they are a novel result for this study, not a “related” or replicating result.

**Figure 5:**
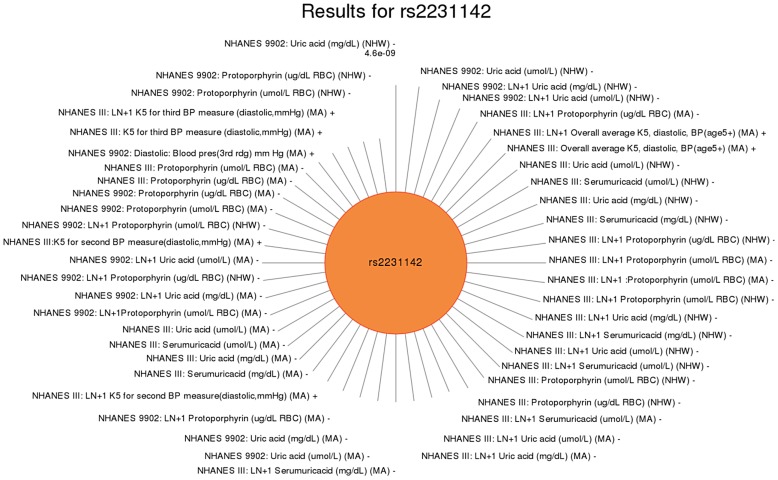
Sun plot of (p<0.01) results for *ABCG* rs2231142, coded allele C. This SNP has been previously reported to be associated with uric acid levels. Significant SNP-phenotype associations (p<0.01) are plotted clockwise with the smallest p-value result at the top. The length of the each line corresponds to the –log(p-value) of each result, with the longest line representing the most significant result for this SNP, meeting our PheWAS replication criteria for inclusion. Study, transformed (LN +1) or untransformed (none) phenotype description, self-reported race-ethnicity, and direction of effect are listed for each association. This SNP was associated with a number of phenotypes in this study including uric acid levels (as previously published) in non-Hispanic whites (NHW) and Mexican Americans (MA), protoporphyrin levels in non-Hispanic whites and Mexican Americans, and diastolic blood pressure in Mexican Americans.

For example, the missense SNP in *ABCG2* rs2231142, also described in novel results, was found to have two novel associations, protoporhyrin (in non-Hispanic whites and Mexican Americans) and blood pressure levels (Mexican Americans), and one replication of a previously known association with uric acid levels (non-Hispanic whites and Mexican Americans). The results for this SNP are plotted in Fig. 5.

**Figure 6:**
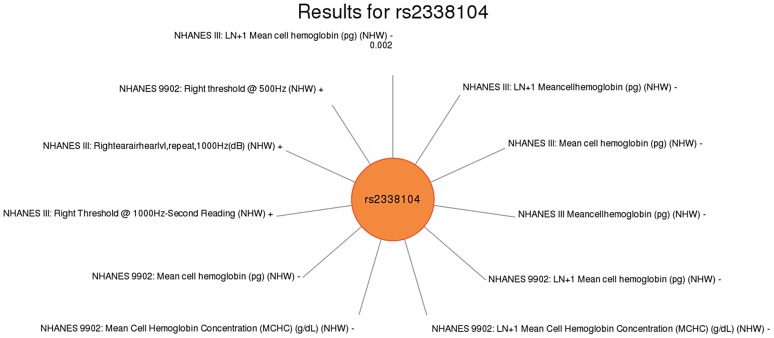
Sun plot of (p<0.01) results for *KCTD10* rs2338104, coded allele G. This SNP was previously associated with HDL-C levels. Significant SNP-phenotype associations (p<0.01) for this study are plotted clockwise with the smallest p-value result at the top. The length of the each line corresponds to the –log(p-value) of each result, with the longest line representing the most significant result for this SNP, meeting our PheWAS replication criteria for inclusion. Study, transformed (LN +1) or untransformed (none) phenotype description, self-reported race-ethnicity, and direction of effect are listed for each association. This SNP was associated with mean cell hemoglobin levels, as well as right ear hearing levels in non-Hispanic whites (NHW).

**Figure 7:**
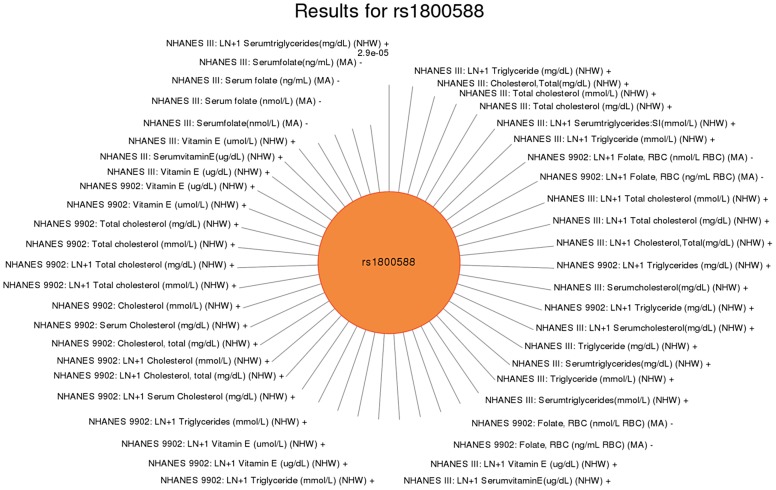
Sun plot of (p<0.01) results for *LIPC* rs1800588, coded allele T. This SNP was previously associated HDL-C in European-descent populations. Significant associations (p<0.01) are plotted clockwise with the most significant value result at the top. The length of the each line corresponds to the –log(p-value) of each result, with the longest line representing the most significant result for this SNP, meeting our PheWAS replication criteria for inclusion. Study, transformed (LN +1) or untransformed (none) phenotype description, self-reported race-ethnicity, and direction of effect are listed for each association. This SNP was associated with a number of phenotypes including folate in Mexican Americans (MA), total cholesterol in non-Hispanic whites (NHW), triglyceride levels in non-Hispanic whites, and vitamin E levels in non-Hispanic whites.

For another example, rs2338104, an intronic SNP in *KCTD10*, which was previously associated with HDL cholesterol (HDL-C) in European-descent populations [17], [25], was associated here with hemoglobin and hearing levels, both novel results in non-Hispanic whites (Fig. 6). Another example of potential pleiotropy was for SNP rs1800588 near *LIPC*, previously associated HDL-C in European-descent populations [15]. We observed significant associations between this SNP and the novel phenotypes of folate (in Mexican Americans) and vitamin E levels (in non-Hispanic whites), as well as replication for cholesterol and the related phenotype of triglycerides (both in non-Hispanic whites; Fig. 7). The intronic SNP rs174547 of *FADS1* provides another example. This SNP was previously associated with phospholipid levels [39], resting heart rate [40], phosphatidylcholine levels [41], HDL-C and triglyceride levels [17] in individuals of European ancestry. Here, this SNP is associated with ferritin levels in Mexican Americans and with folate levels in non-Hispanic blacks.

To further characterize these putative pleiotropic relationships, we compared and contrasted direction of effect for each association (Table 4). We found variants related to potentially protective effects for certain traits, and a potential risk effects for other traits. For example, intergenic SNP rs12678919 near *LPL* was associated with HDL cholesterol levels in non-Hispanic whites with a positive direction of effect and hearing in non-Hispanic blacks with a negative direction of effect (coded allele G). Intronic SNP rs174547 in *FADS1* was associated with ferritin levels in Mexican Americans with a positive direction of effect and folate (in non-Hispanic blacks) and triglycerides (in non-Hispanic whites) with a negative direction of effect (coded allele T). The intronic SNP rs6855911 in *SLC2A9* was associated with uric acid (in both non-Hispanic blacks and Mexican Americans) with a negative direction of effect and thigh circumference measurements (non-Hispanic blacks) with a positive direction of effect (coded allele G).

### Investigating Interrelationships within PheWAS Results

PheWAS-significant results provide an opportunity to explore the relationships between SNPs, genes, traits/outcomes, and pathways or other known relationships between genes and gene-products. We used the software tool Biofilter to identify the genes the PheWAS-significant SNPs were within or closest to. We then used Biofilter to annotate the resultant genes using the Kyoto Encyclopedia of Genes and Genomes (KEGG) [42], Gene-Ontology (GO) [43], and NetPath [44] which allowed us to identify any known connections between genes due to shared biological pathways or other known biological connections. After stratifying the results by race-ethnicity, we used Cytoscape [45] to visualize the connections between genes based on their annotation. We present here the networks where there were two or more SNPs significant in our PheWAS connected via genes and those two or more genes were connected by a pathway or other gene-gene connection.

**Figure 8:**
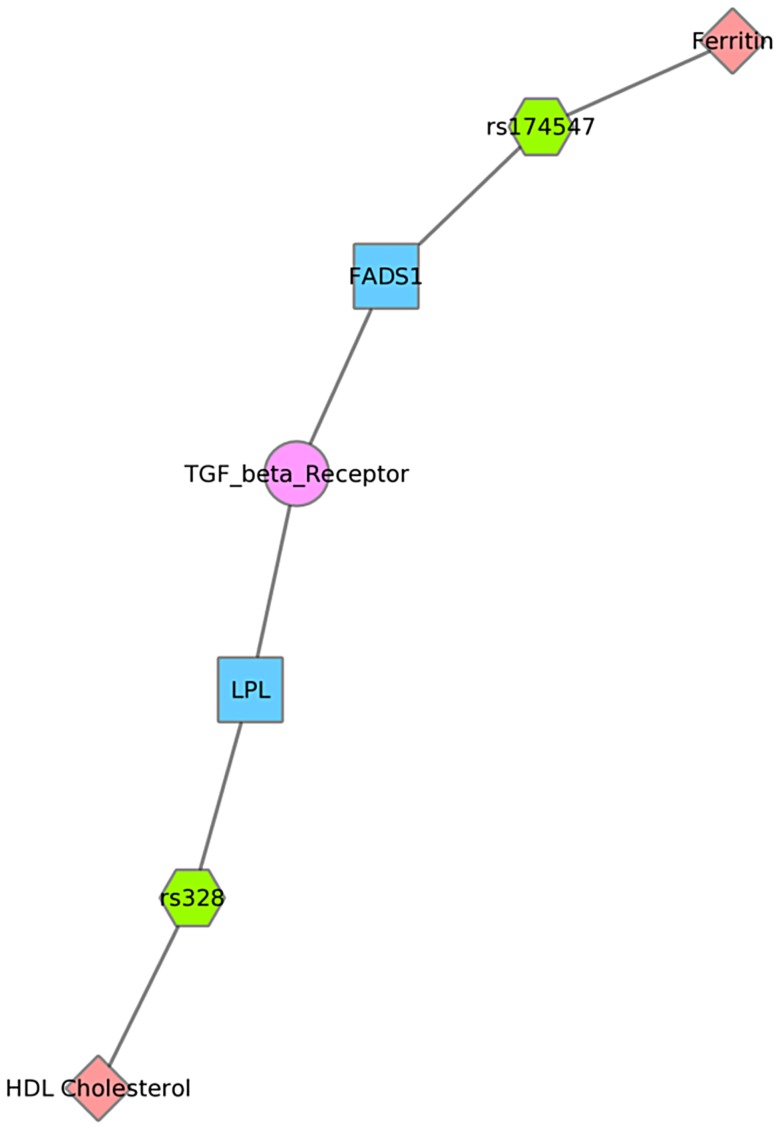
Using PheWAS results, Biofilter, and Cytoscape to explore gene-gene connections with NetPath. We used Biofilter to annotate the SNPs of this PheWAS with gene information. We then mapped the genes to concomitant pathways or other gene groupings through GO, KEGG, and NetPath. This is one example for the results for Mexican Americans and annotation with NetPath. The pink diamonds are associated phenotypes of this PheWAS, the green hexagons are SNPs, blue boxes are genes, and circles are biological connections that link genes together, in this case the two genes are in the same TGF NetPath biological pathway. Thus, we see that in the PheWAS results, the *LPL* SNP rs328 had a significant association with HDL cholesterol levels, and *FADS1* rs17547 association with Ferritin levels, and both genes are found in the TGF beta receptor pathway.

**Figure 9:**
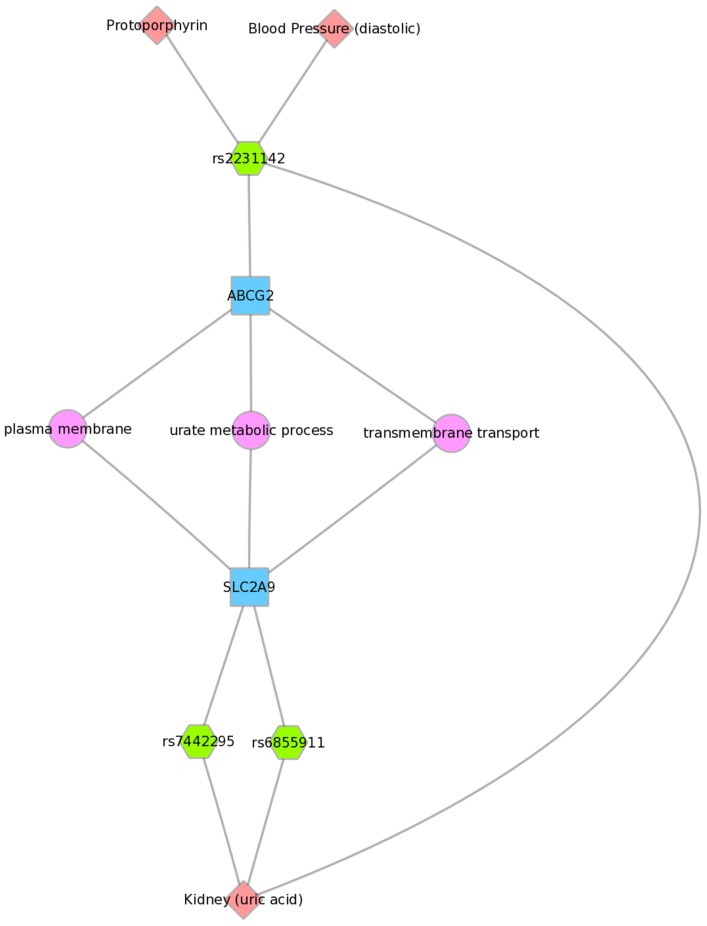
Using PheWAS results, Biofilter, and Cytoscape to explore gene-gene connections with GO biological processes. Three SNPs were associated with uric acid levels in Mexican Americans: rs2231142, rs7442295, rs685911 (green hexagons). One of the SNPs is within the gene *ABCG2*, and the other two SNPs are within *SLC2A9* (blue boxes). Both *ABCG2* and *SLC2A9* are found within the GO biological process “urate metabolic process”, a collection of the gene products involved in the chemical reactions and pathways involving urate. This was also found for non-Hispanic whites.

For example, Fig. 8 shows one example for PheWAS results in Mexican Americans, where *LPL* SNP rs328 had a significant association with HDL-C levels, and the *FADS1* SNP rs17547 had an association with ferritin levels. Both genes are found in the TGF-β receptor regulated NetPath pathway. Fig. 9 shows another example in Mexican Americans in which three SNPs were associated with uric acid levels: rs2231142, rs7442295, rs685911. One of the SNPs is located within the gene *ABCG2*, and the other two SNPs are located within *SLC2A9* (blue boxes). Both *ABCG2* and *SLC2A9* are found within the GO biological process “urate metabolic process”, a collection of the gene products involved in the chemical reactions and pathways involving urate. These same connections were also found for non-Hispanic whites, as this group had a PheWAS-significant association between these SNPs and uric acid levels. One of the SNPs, rs2231142, was also associated with diastolic blood pressure and protoporphyrin levels.

**Figure 10:**
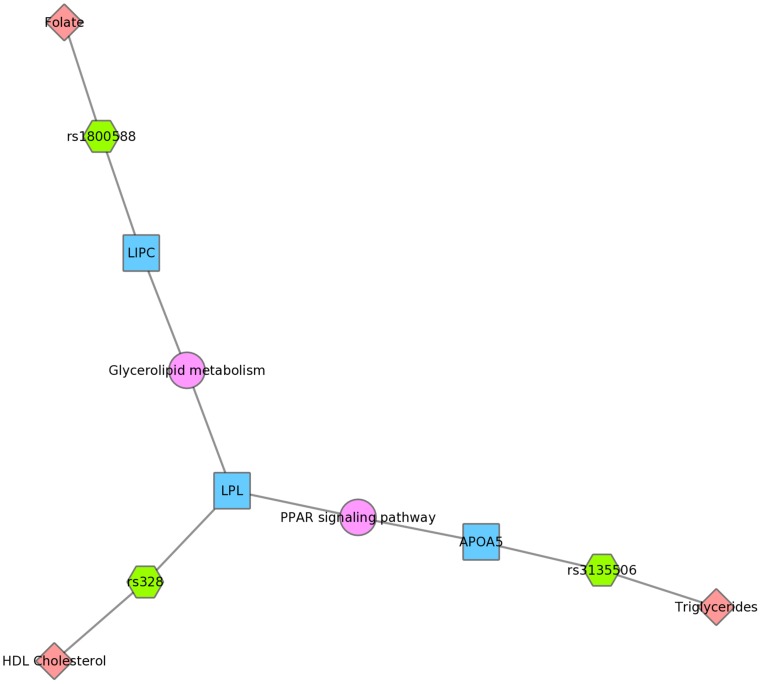
Using PheWAS results, Biofilter, and Cytoscape to explore gene-gene connections with KEGG connections. The *LIPC* SNP rs1800588 was associated with folate levels, the *LPL* SNP rs328 was associated with HDL cholesterol, and both of these genes are in the glycerolipid metabolism KEGG pathway in Mexican Americans. The *APOA5* SNP rs135506, associated with triglyceride levels in our study, shares the PPAR signaling pathway along with *LPL*.

Fig. 10 displays an example using KEGG and the Mexican American PheWAS results. *LPL* and *LIPC* both are involved in the KEGG biological process “glycerolipid metabolism”. *LPL* SNP rs328 was associated in this study with HDL-C, while *LIPC* SNP rs1800588 was associated with folate levels. *LPL* was also involved in the KEGG pathway “Peroxisome Proliferator-Activated Receptor (PPAR) signaling pathway”, along with *APOA5*, which was associated with triglyceride levels through its SNP rs3135506. PPARs are transcription factors activated by lipids.

## Discussion

For this PheWAS, performed using the data of NHANES, we have replicated a number of previously published results and have found novel and pleiotropic associations. For example, for rs2231142, a missense SNP in *ATP-binding cassette subfamily G member 2 (ABCG2)*, we replicated previous associations with uric acid levels observed in European-descent populations and in Mexican Americans with the same direction of effect. Additionally, we identified a novel association for this SNP with protoporphyrin in both the European-descent population and Mexican Americans, where the coded allele (C) was associated with increased uric acid levels as well as increased protoporphyrin. This PheWAS finding is intriguing in light of some of the known connections that link protoporhyrin with uric acid levels, suggesting the potential for this SNP to have an impact on the levels of one or both resulting in the associations identified here. Protoporhyrin combines with heme to form iron-containing proteins. This gene is in the bile secretion pathway [42], and bile consists of substances including bilirubin, which is converted from heme/porphyrin [43]. Thus, the observed association is consistent with a known biological process. There is also a known correlation between ferritin levels and uric acid levels, and urate forms a coordination complex with iron to diminish electron transport, acting as an iron chelator and antioxidant [46]. This correlation implies an expected link between protoporphyrin and uric acid association results; however, we did not observe an association with ferritin levels in this study for this SNP.

The PheWAS significant association between rs2231142 and blood pressure levels was only observed in Mexican Americans. However, the direction of effect is opposite as seen for uric acid levels and protoporphyrin. There is a demonstrated positive correlation between high blood pressure and high serum uric acid levels [44], [45], but the relationships between rs2231142 and diastolic blood pressure compared with serum uric acid levels in our study were inconsistent, suggesting an independent relationship between this SNP and the two phenotypes. Thus, this is an example of the novel discoveries that can occur with the PheWAS approach that would not be found through only investigating the association between multiple SNPs and a single trait outcome or phenotype.

Another intriguing result was for rs2338104, an intronic SNP in the *potassium channel tetramerisation domain containing 10 (KCTD10)* gene, which is a member of the polymerase delta-interacting protein 1 gene family. *KCTD10* has been previously associated with DNA synthesis/cell proliferation [46], HDL cholesterol levels [13], [21], and interaction with an ubiquitin ligase [47]. In this study, *KCDT10* rs2338104 was associated with right ear hearing levels and mean cell hemoglobin levels in non-Hispanic whites. The biological function of *KCDT10* has not been extensively studied; consequently, biological explanations for the relationship between this variant and hearing or mean cell hemoglobin do not yet exist.

Novel associations for hematologic traits were found in this PheWAS. The SNP rs1800795 near gene *interleukin 6* (*IL6*) and rs4355801 in *tumor necrosis factor receptor superfamily, member 11b (TNFRSF11B)* had significant association with white blood cell counts in non-Hispanic blacks. There are known associations between hematologic traits and genetic variants on chromosome 1 in African Americans, spanning a wide region of chromosome 1 [47]. This region of association is due to the presence of the African-derived Duffy Null polymorphism, a genetic variant protective against Plasmodium vivax malaria. Presence of this variant explains the lower white blood cell and neutrophil counts in African Americans [48]. However, neither rs1800795 nor rs4355801 are located on chromosome 1 and therefore represent potentially unique associations with hematologic traits.

Further novel associations with circulating vitamin levels were found. The SNP rs1260326 was associated with vitamin A in non-Hispanic whites. Vitamin E was associated with rs13266634, rs28927680, and rs1800588 in non-Hispanic whites and rs964184 in non-Hispanic whites and Mexican Americans. Additionally, folate levels were associated with rs174547 in non-Hispanic blacks and rs1800588 in Mexican Americans. When considering the direction of effect for the vitamin levels, we found that rs174547, an intronic SNP in *fatty acid desaturase 1 (FADS1)*, was associated with ferritin and iron levels with different direction of effect in Mexican Americans. Conversely, vitamin E showed the same direction of effect as triglycerides. Recent findings indicate a potential relationship between vitamin E intake and triglyceride levels for certain SNPs [49]. Thus, these results may be reflective of an interaction between variability in vitamin E intake and genetic variance.

Other SNPs with pleiotropic effects showed associations with different directions of effect. For example, rs780094 in the intron of *glucokinase regulator (GCKR)* was associated with serum glucose levels with a positive direction of effect (0.67) and potassium and vitamin B6 intake levels with a negative direction of effect (β = −0.05 and −0.11, respectively) in Mexican Americans. This result is consistent with the demonstrated inverse relationship between potassium intake and glucose intolerance [50]. Likewise, glucose tolerance has been found to increase upon vitamin B6 supplement intake in women with gestational diabetes mellitus [51], [52]. One possibility, requiring further investigation, is that this SNP modulates the effect of vitamin B6 and potassium on glucose levels.

Fourteen of our results showed both a significant PheWAS association and the same direction of effect for a different race-ethnicity. We did not investigate non-significant results with a similar direction of effect for this study. We evaluated the differences in allele frequency across the two surveys, across race-ethnicity, for the SNPs that met our criteria for PheWAS replication (S5 Table). There were not consistent trends between similar or markedly different allele frequencies and whether we did or did not see the same SNP-phenotype associations across more than one race-ethnicity. The reason for differences in association may lie in the variation between linkage disequilibrium patterns across populations. Additionally, as genetic architecture can vary across different race-ethnicities, there is the potential for finding novel associations that exist in only one population. Low power due to sample size could have also contributed to fewer significant associations in non-Hispanic black and Mexican American populations, when compared to non-Hispanic whites, as the sample sizes were generally smaller. Further, phenotypic outcome is impacted by both genetic variation and environmental exposure variation, and thus some associations may not replicate across race-ethnicity in part due to potentially different environmental exposure across racial/ethnic groups. Also, there are differences in the median age across race-ethnicity for the two surveys that could contribute to being unable to detect SNP-phenotype associations across different race-ethnicities.

We found examples of gene-gene connections that link our PheWAS results from the SNP to gene to pathway level. These examples show the utility of applying known information about genes to provide biological context for individual PheWAS results through visually linking the information together. Multiple connections not readily apparent when exploring tabular results can be highlighted with this approach. For example, Fig. 9 shows three SNPs within two different genes that are within the GO biological process of “urate metabolic process”, a group of gene products involved in the chemical reactions and pathways involving urate. These SNPs are all associated with uric acid levels in our PheWAS. These SNPs have previously reported associations with uric acid levels, and these genes are known to be involved with pathways that contain urate. However, through connecting phenotypes, SNPs, genes, and pathways, and visualizing the results, we can more clearly show how single genetic variants are likely biologically linked to outcome variation. Further, this example shows the SNP rs2231142 associated with two other phenotypes, as described earlier in this discussion.

We also presented network results in Figs. 8, 9 and 10. The results presented in Fig. 8 show two SNPs in different genes that both are found in the TGF-β receptor regulated NetPath pathway. This would not have been evident in the PheWAS without applying annotation from known pathways. Fig. 10 shows one example of two genes involved in the KEGG biological process “glycerolipid metabolism”. Here, one SNP is associated with HDL-C levels, and, interestingly, a separate SNP in the network is associated with folate levels. Plasma folate levels have been associated with lipoprotein profiles [49]. Further, the *LPL* SNP rs328 was associated in this study with HDL-C and is also involved in the KEGG pathway “Peroxisome Proliferator-Activated Receptor (PPAR) signaling pathway”, along with a SNP in *APOA5*, which was associated with triglyceride levels. PPARs are transcription factors activated by lipids. In the future we will continue to use this network approach, to highlight both the biological context that supports results found in PheWAS and the biological annotation that may identify relationships that forge new hypotheses about the connection between genetic variation and complex outcomes.

One limitation to the current PheWAS approach is the risk of false-positive associations due to the large number of tests for association between SNPs and phenotypes. For this analysis, we required replication of association results across NHANES to reduce the type-1 error rate. Correcting for multiple hypothesis testing to account for the comprehensive associations in PheWAS, and thus potentially inflated Type I error, based on the number of tests/studies/groups can be problematic for multiple reasons. Most multiple testing calculations assume independent tests, which we do not have here as phenotypes are correlated across our PheWAS studies. Also, our power from one result to another can vary in part due to variations in sample size for the specific phenotype. In addition we used phenotype-class binning of results which results in different numbers of sub-phenotypes in each bin for potential replication. Future work includes research into identifying additional methods for multiple testing burden in PheWAS, such as permutation testing. Another limitation to the PheWAS approach is the high-throughput nature of the analysis. For instance, adjustments were not made for participants on medication that could modify or lower measurements such as lipids. The results are considered preliminary and bear further inquiry. However, it is notable that we observed replication of a number of previously published results with the same direction of effect indicating that our high-throughput approach is functional for a number of measures. Because we chose to seek replication across NHANES surveys, we did not explore results unique to any one survey.

A major strength of the PheWAS approach is the potential for novel discoveries about genetic variants and their relation to phenotypes for future investigation as well as to replicate results found in GWAS. Phenome-wide associations provide the opportunity to uncover complex networks of phenotypes involved in disease through tests of association between genetic variants and a broad range of phenotypes. Utilizing existing epidemiologic collections such as the diverse NHANES allows for potential generalization of variant-phenotype relationships across race-ethnicities.

We have found novel associations for phenotypes such as white blood cell count and vitamin levels for SNPs with different previously known associations. We also have found indications of pleiotropy. Further, because this approach investigates single SNPs with multiple phenotypes, results with contrasting direction of effect can be investigated. We explored the results of this PheWAS within the context of additional biological information including the use of network diagrams. In addition, we were able to pursue this across multiple race-ethnicities, whereas much of the approach in GWAS has been within European Americans. The results described here demonstrate the utility of the PheWAS approach to expose relevant results that contrast what is known about the relationships between multiple phenotypes and between genotype and phenotype to uncover the complex nature of human traits.

## Materials and Methods

### Study Design and Populations

Two NHANES surveys [53] were included in the PheWAS analyses. The epidemiological survey data and DNA samples of NHANES III were collected between 1991–1994 and Continuous NHANES was collected between 1999–2000 and 2001–2002. For some of the phenotypes, harmonization across NHANES III and Continuous NHANES was possible. Thus, for a subset of phenotypes, we were able to use the two surveys combined in analyses we refer to as NHANES *Combined*. NHANES measures the health and nutritional habits of U.S. participants regardless of health status across race-ethnicity, by collecting medical, dietary, demographic, laboratory, lifestyle, and environmental exposure data via questionnaire, direct laboratory measures, and a physical exam. In NHANES, specific age groups (such as the young elderly) and racial/ethnic groups are oversampled. The epidemiological data of NHANES and the associated DNA samples were collected by the National Center on Health Statistics (NCHS) at the Centers for Disease Control and Prevention (CDC). All procedures were approved by the CDC Ethics Review Board and written informed consent was obtained from all participants. Because no identifying information is available to the investigators, Vanderbilt University's Institutional Review Board determined that this study met the criteria of “non-human subjects.”

### Genotyping and SNP Selection

For this study, EAGLE genotyped 80 GWAS-identified variants in two NHANES datasets representing three surveys: *NHANES III*, collected between 1991 and 1994, and *Continuous NHANES*, collected between 1999–2000 and 2001–2002. The majority of the SNPs within our study were chosen for genotyping based on published lipid trait genetic association studies. Also included in this study are SNPs previously associated with a range of other phenotypes, and we detail information about these SNPs in S1 Table, including the genotyping method for each SNP (unless the SNP was already available within NHANES before EAGLE genotyping, and there we cite the lab that provided the genotypic data to NHANES). Genotyping was performed in a total of 14,998 NHANES participants with DNA samples including 6,634 self-reported non-Hispanic whites, 3,458 self-reported non-Hispanic blacks, and 3,950 self-reported Mexican Americans. Genotypes included in this study were accessed from (1) genotyping performed using Sequenom by the Vanderbilt DNA Resources Core, or (2) existing data in the Genetic NHANES database. In addition to genotyping experimental NHANES samples, blinded duplicates provided by CDC and HapMap controls (n = 360) as part of the PAGE study were also genotyped. Quality control, which included concordance and Hardy Weinberg Equilibrium, was performed on all SNPs by the CDC. All SNPs that passed quality control are available for secondary analyses through NCHS/CDC.

### Statistical Methods

Single SNP unadjusted tests of association were performed for 80 SNPs available in NHANES III and Continuous NHANES and 1,008 phenotypes. When the exact phenotype was measured in NHANES III and Continuous NHANES, the unadjusted tests of association were also performed for all samples as part of Combined NHANES. As outlined in the PAGE Study [7] tests of association between all SNPs and phenotypes were performed using linear or logistic regression, depending on whether the phenotype was binary or continuous. For categorical phenotypes, binning was used to create new variables of the form “A versus not A” for each category, and logistic regression was used to model the new binary variables. All continuous phenotypes were natural log transformed, following a y to log (y+1) transformation of the response variable with +1 added to all continuous measurements before transformation to prevent variables recorded as zero from being omitted from analysis. All analyses were stratified by self-reported race-ethnicity. Analyses were performed remotely in SAS v9.2 (SAS Institute, Cary, NC) using the Analytic Data Research by Email (ANDRE) portal of the CDC Research Data Center in Hyattsville, MD.

### NHANES Phenotypes

A wide range of phenotypic variables was available for both NHANES III and Continuous NHANES. We used only phenotypes for this study that could be binned into phenotype classes across more than one NHANES (see phenotype classes section for more details), so that we could seek replication for association results across surveys. The phenotypes of this study are listed in S6 Table. Detailed information on the collection of each of the phenotypes is available through the CDC, for NHANES III (http://www.cdc.gov/nchs/nhanes/nh3data.htm) and for Continuous NHANES (http://wwwn.cdc.gov/nchs/nhanes/search/nhanes_continuous.aspx)

### Phenotype Classes

To facilitate comparisons across NHANES, similar phenotypes from each of the NHANES were binned into 184 “phenotype-classes” (Table 2) via manual inspection of one person and reviewed by a second individual, similar to the phenotype binning of [4]. The development of phenotype-classes was necessary for several reasons. First, not all phenotypes and exposures were surveyed or collected in the same way for each iteration of NHANES, and thus could not be completely harmonized. However, some of these phenotypes were similar enough across surveys and to be binned into the same phenotype-class (for example, “Arm Circumference” and “Upper Arm Length” were both binned in the “Body Measurements (Arm)” phenotype-class). Second, when matching phenotypes and exposures, the labels across and within NHANES vary even for the same phenotypes. For example “Vitamin A” and “Serum Vitamin A” both measured the same phenotype and thus were both classified in the “Vitamin A” phenotype-class. For the majority of PheWAS results, there were multiple significant NHANES measures for each phenotype class, and we reported the lowest p-value in descriptions of the PheWAS results within the figures and the results. Our list of the phenotypes of this study also includes their respective phenotype class, listed in S6 Table.

### Threshold of Significance

A significant PheWAS result met all of the following criteria: 1) a SNP-phenotype association was observed in both NHANES III and Continuous NHANES, 2) with p-value <0.01, 3) allele frequency >0.01, 4) sample size >200, 5) for the same race-ethnicity, 6) phenotype class, and 7) direction of effect. For each of these consistent associations, we examined tests of association results for Combined NHANES. Significant PheWAS results were then plotted using Phenogram [50] and PheWAS-View[51], software specifically developed for visualization of PheWAS results (http://ritchielab.psu.edu/ritchielab/software/). The expanded results for all 69 results meeting our PheWAS significance criteria are presented in S2 Table.

### Correlations between Phenotypes

We calculated pairwise Pearson correlations between all phenotypes that had a significant PheWAS result, for NHANES III and Continuous NHANES, stratified by race-ethnicity. For any significant PheWAS phenotype, we listed correlations for any phenotypes with a correlation >0.6 with the significant PheWAS phenotype list.

We took the absolute value of the correlations and used the statistical package R [52] to create a clustered heat map of the correlations with color ranging from light yellow to dark blue. We present our correlation matrices in S1–S6 Figures. The most correlated phenotypes are shown in a light yellow color, the less correlated a phenotype pair, the more blue on the heatmap.

### Biofilter

Biofilter [53], [54] is a software package that allows the user to download and automatically integrate several different knowledge databases into a single accessible database called the Library of Knowledge Integration, and then run queries via Biofilter with the resultant integrated data (https://ritchielab.psu.edu/ritchielab/software/). We used Biofilter to annotate the SNPs of this study with the location and identification of the nearest genes to each of our SNPs, from NCBI dbSNP and NCBI Gene (Entrez) (http://www.ncbi.nlm.nih.gov/). We also applied information from the Kyoto Encyclopedia of Genes and Genomes (KEGG) [42], Gene Ontology (GO) [43], and NetPath [44]. This allowed us to highlight known connections between genes. Thus, we were able to identify any biological pathway or grouping connections between the genes SNPs were in or near in our study.

### Cytoscape

After we used Biofilter to annotate the genes as described above, we stratified the results by race-ethnicity. We used Cytoscape [45] to visualize the connections between genes based on their annotation. Using this visualization tool, we explored networks where one or more SNPs were connected, via genes, to mutual pathways or genes, and we did not further investigate any resultant networks comprised of single SNPs.

### RegulomeDB

RegulomeDB [55] was used to annotate PheWAS-significant SNPs in this study with functional and regulatory information for our analyses. The results of this analysis are included in Table 4.

## Supporting Information

S1 Figure Heatmap of correlations for phenotypes in NHANES III Non-Hispanic blacks (NHB).
(PNG)

S2 Figure Heatmap of correlations for phenotypes in NHANES III Non-Hispanic whites (NHW).
(PNG)

S3 Figure Heatmap of correlations for phenotypes in NHANES III Mexican Americans (MA).
(PNG)

S4 Figure Heatmap of correlations for phenotypes in Continuous NHANES Non-Hispanic blacks (NHB).
(PNG)

S5 Figure Heatmap of correlations for phenotypes in Continuous NHANES Non-Hispanic Whites (NHW).
(PNG)

S6 Figure Heatmap of correlations for phenotypes in Continuous NHANES Mexican Americans (MA).
(PNG)

S1 Table Information on the 80 SNPs of this study.
(XLSX)

S2 Table All results of this study meeting our PheWAS criteria for replication.
(XLSX)

S3 Table Results of this study replicating previously published associations.
(XLSX)

S4 Table Results of this study highly related to previously published associations.
(XLSX)

S5 Table Comparing the difference in allele frequency across race-ethnicity within this study.
(XLSX)

S6 Table Phenotypes and phenotype classes of this study.
(XLSX)
