# Supplementary figures and images for: Detection of Pleiotropy through a Phenome-Wide Association Study (PheWAS) of Epidemiologic Data as Part of the Environmental Architecture for Genes Linked to Environment (EAGLE) Study
Source: PLoS Genet. 2014 Dec 4;10(12):e1004678. doi: 10.1371/journal.pgen.1004678 (PMC4256091; doi:10.1371/journal.pgen.1004678)

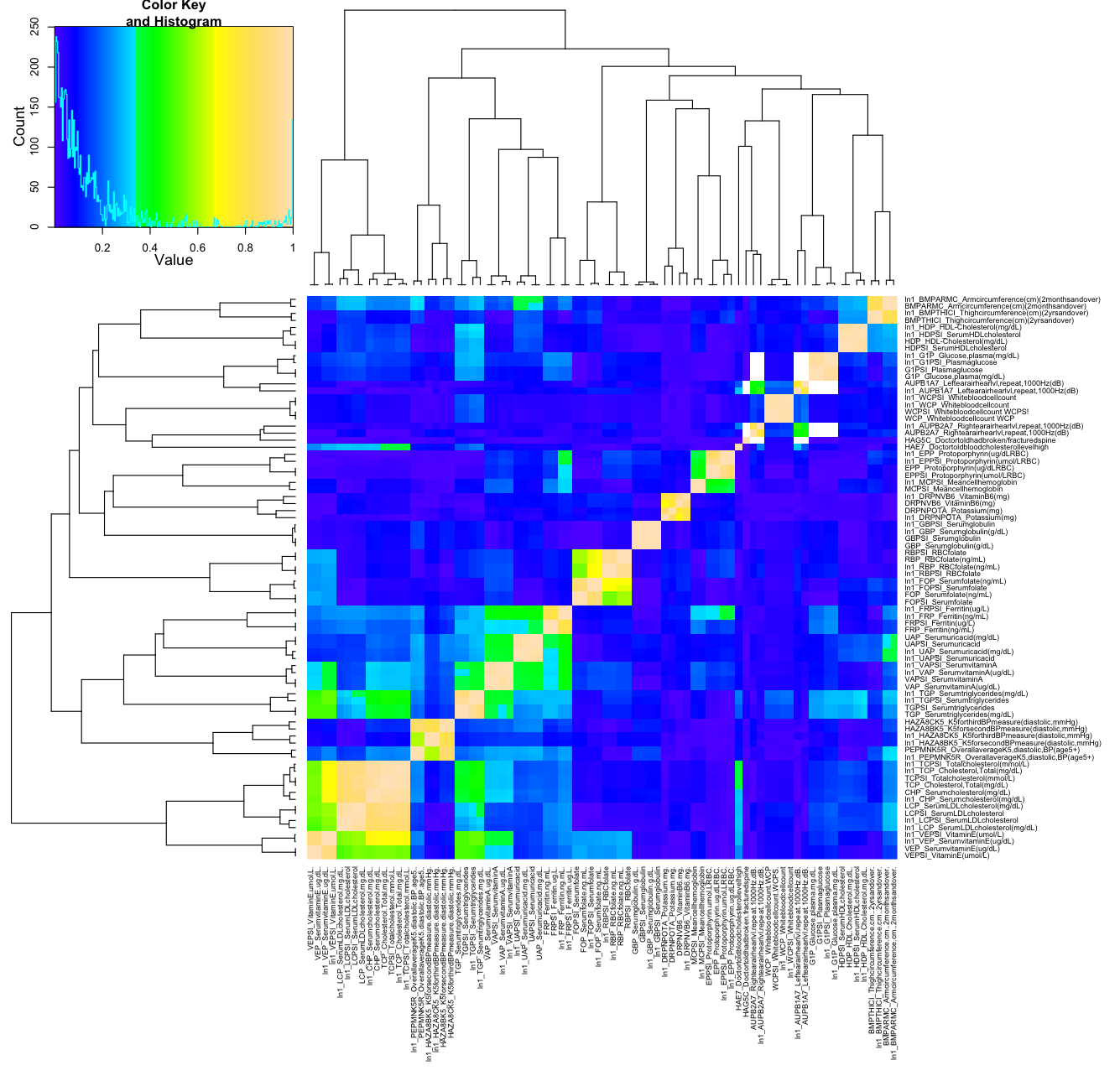

Supplement: S1 Figure — Heatmap of correlations for phenotypes in NHANES III Non-Hispanic blacks (NHB). (PNG) [file pgen.1004678.s001.png]

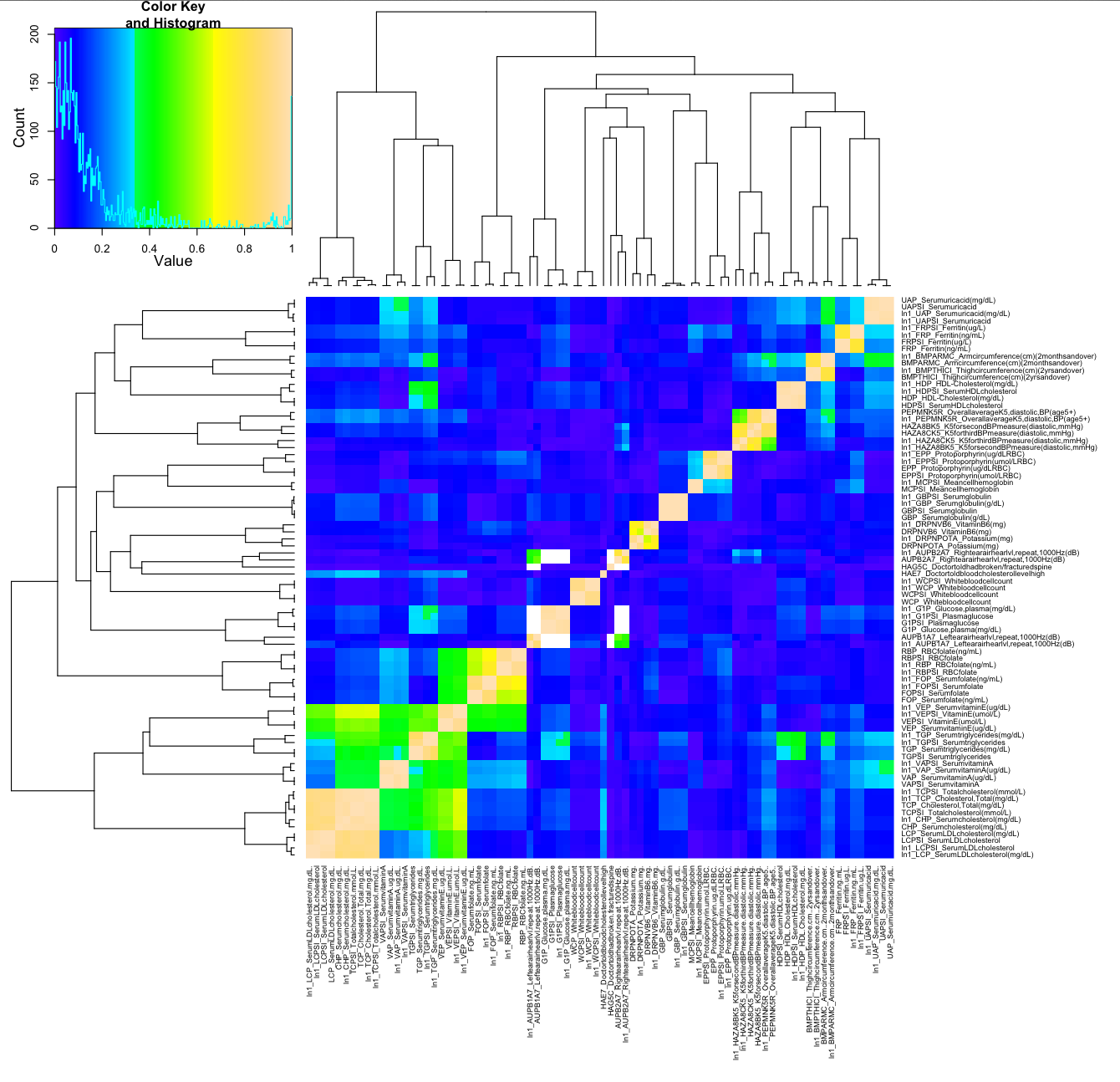

Supplement: S2 Figure — Heatmap of correlations for phenotypes in NHANES III Non-Hispanic whites (NHW). (PNG) [file pgen.1004678.s002.png]

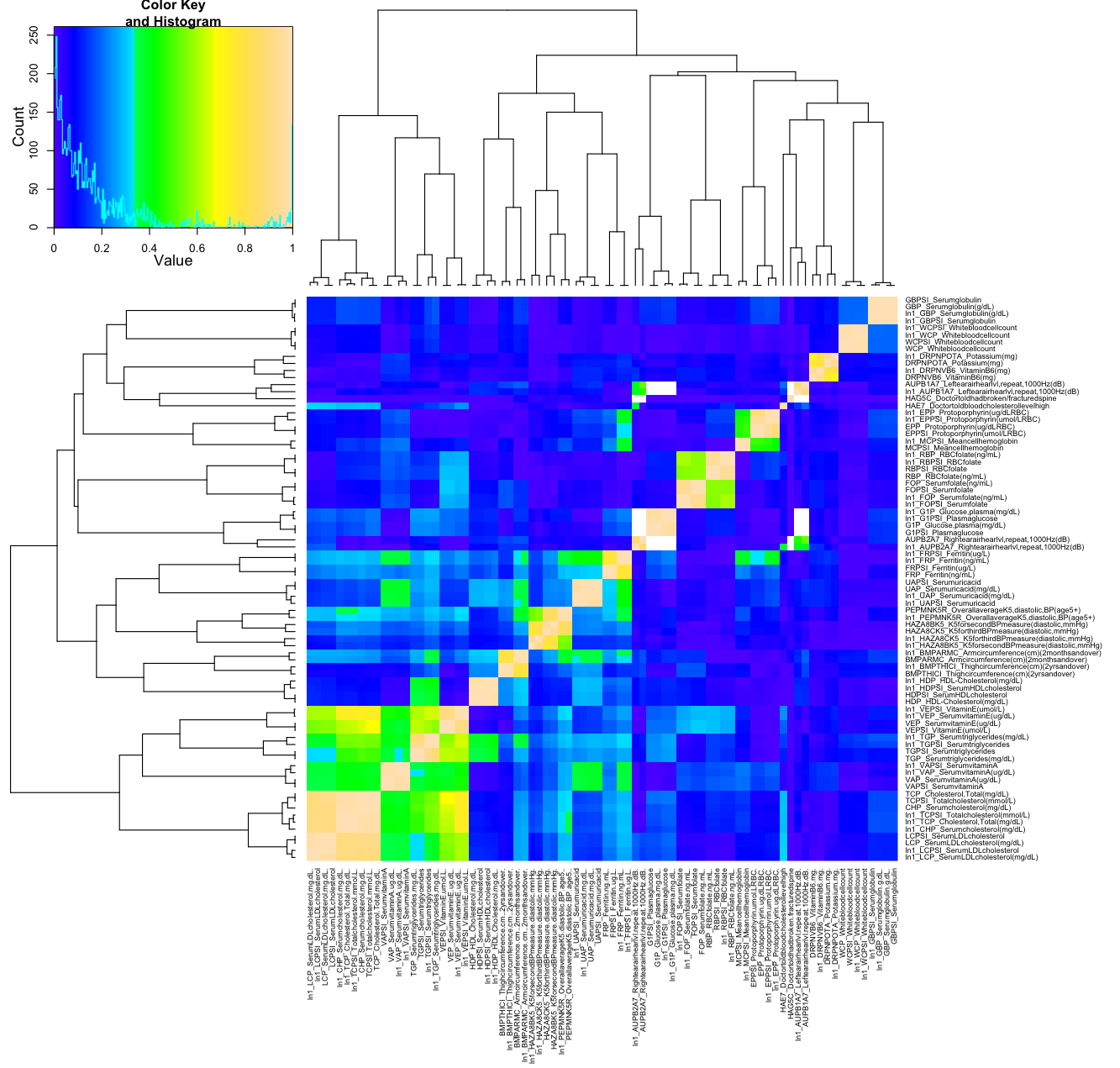

Supplement: S3 Figure — Heatmap of correlations for phenotypes in NHANES III Mexican Americans (MA). (PNG) [file pgen.1004678.s003.png]

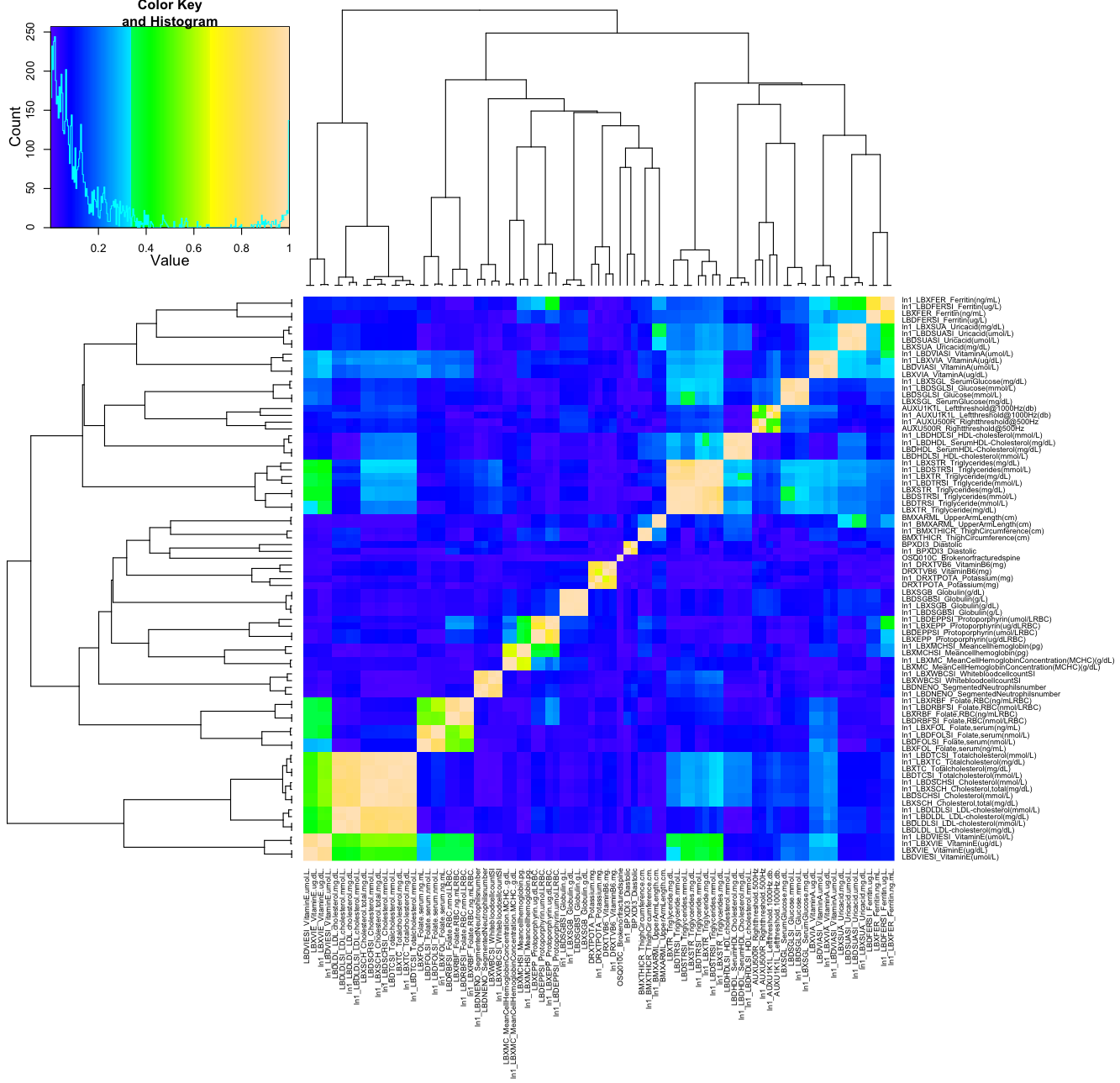

Supplement: S4 Figure — Heatmap of correlations for phenotypes in Continuous NHANES Non-Hispanic blacks (NHB). (PNG) [file pgen.1004678.s004.png]

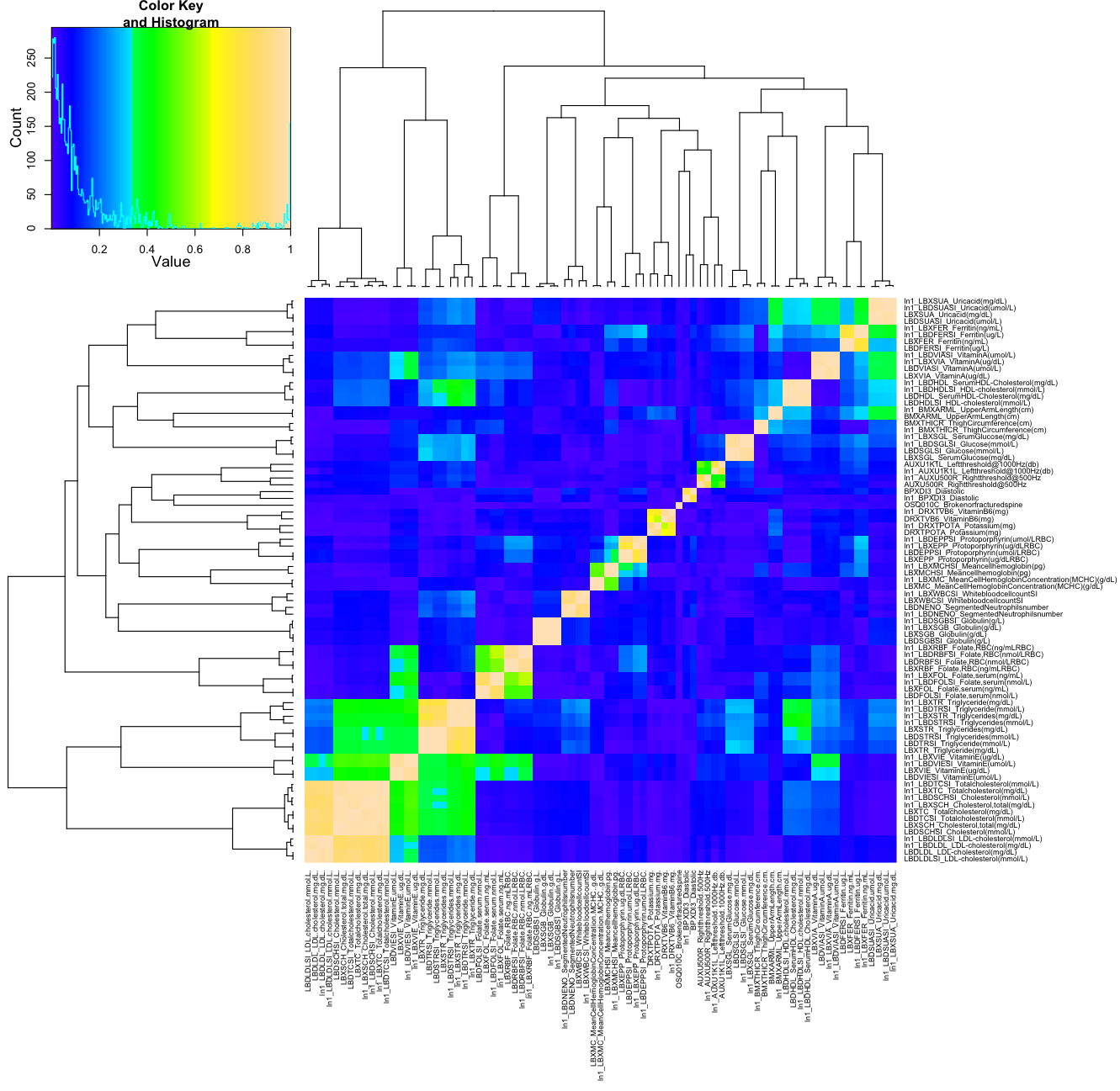

Supplement: S5 Figure — Heatmap of correlations for phenotypes in Continuous NHANES Non-Hispanic Whites (NHW). (PNG) [file pgen.1004678.s005.png]

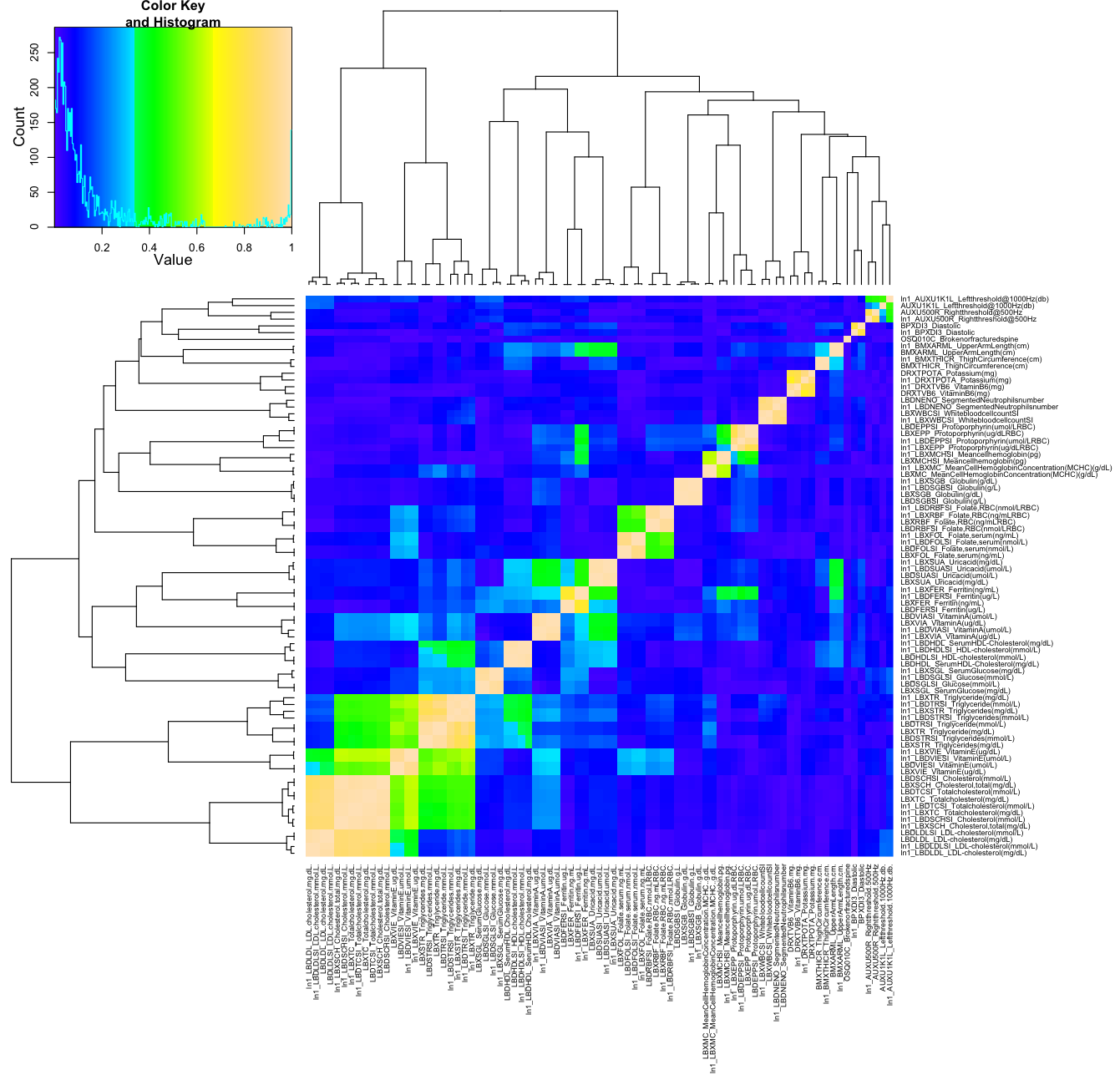

Supplement: S6 Figure — Heatmap of correlations for phenotypes in Continuous NHANES Mexican Americans (MA). (PNG) [file pgen.1004678.s006.png]
